# The Role of Solute Carrier Transporters in Efficient Anticancer Drug Delivery and Therapy

**DOI:** 10.3390/pharmaceutics15020364

**Published:** 2023-01-21

**Authors:** Elena Puris, Gert Fricker, Mikko Gynther

**Affiliations:** Institute of Pharmacy and Molecular Biotechnology, Ruprecht-Karls-University, Im Neuenheimer Feld 329, 69120 Heidelberg, Germany

**Keywords:** cancer, solute carrier transporters, drug resistance

## Abstract

Transporter-mediated drug resistance is a major obstacle in anticancer drug delivery and a key reason for cancer drug therapy failure. Membrane solute carrier (SLC) transporters play a crucial role in the cellular uptake of drugs. The expression and function of the SLC transporters can be down-regulated in cancer cells, which limits the uptake of drugs into the tumor cells, resulting in the inefficiency of the drug therapy. In this review, we summarize the current understanding of low-SLC-transporter-expression-mediated drug resistance in different types of cancers. Recent advances in SLC-transporter-targeting strategies include the development of transporter-utilizing prodrugs and nanocarriers and the modulation of SLC transporter expression in cancer cells. These strategies will play an important role in the future development of anticancer drug therapies by enabling the efficient delivery of drugs into cancer cells.

## 1. Introduction

Cancers accounts for nearly 10 million deaths worldwide yearly, with lung, liver, stomach and breast cancers currently being the most common causes of cancer death worldwide [1]. It has been estimated that the yearly cancer deaths will increase to 16 million by the year 2040, indicating the growing need for the development of anticancer treatments [2]. Advances in cancer research have increased our knowledge of cancer biology, led to identification of new molecular targets and facilitated the development of new anticancer treatments. Among the anticancer treatment approaches, such as surgical intervention, radiation therapy and laser therapy, drug therapy is a common option for cancer treatment [3]. In addition, unlike the classic chemotherapy strategy, which affects both normal cells and cancer cells, there have been significant advances during the previous decades in the development of targeted therapies, which interfere with the altered key oncogenes or tumor suppressor genes involved in tumor promotion [4]. These targeted therapies include, for example, tyrosine kinase inhibitors and drugs targeting KRAS G12C mutations. However, targeted therapies may not always be effective, as the tumors may not express the target protein or have the targeted mutation. In addition, targeted therapies may lose their efficacy during the drug treatment. Therefore, chemotherapy still has a role in anticancer treatment. The development of drug resistance to chemotherapy and molecularly targeted therapies results in the non-responsiveness of a large number of patients to multiple functionally and structurally diverse anticancer drugs, also known as the “multidrug resistance” (MDR) phenomenon [5]. The MDR phenomenon and the resulting ineffectiveness of anticancer drug treatment is estimated to cause over 90% of the cancer-related deaths in patients with metastatic cancer [6]. Therefore, the elucidation of the drug resistance mechanisms and development of the strategies to overcome this complex issue is paramount for the development of successful anticancer drug therapies.

There are several mechanisms of anticancer drug resistance, and these mechanisms can occur in cancer cells sequentially or concurrently [7]. One of the mechanisms underlying resistance to anticancer agents is the limited tumor accumulation of the drugs. In this respect, membrane transporters are responsible for the efflux (mainly the members of the ATP-binding cassette, or ABC, transporter family) and influx (mainly the members of the solute carrier, or SLC, transporter family) of drugs across the cellular membrane. Thereby, transporters regulate drug concentrations at the target site in cancer cells and healthy tissues, affecting clinical outcomes. To date, most studies have focused on investigating the mechanisms underlying the ABC transporter-mediated chemoresistance associated with the up-regulation of several ABC transporters (e.g., ABCB1, ABCG2 and ABCC1) in response to anticancer drugs [8,9,10]. In contrast, knowledge of the role of the SLC transporters, which are key players in drug and nutrient cellular uptake to cancer cells, in anticancer drug resistance is limited [11]. As a result, the majority of the research on overcoming anticancer drug resistance due to limited drug accumulation has been focused on the development of efflux transporter inhibitors. However, a growing knowledge of SLC transporters and their role in anticancer drug resistance opens new horizons for the utilization of SLCs to improve anticancer drug delivery, and it has become evident that more efforts should be made in the development of influx-transporter-targeting drug delivery strategies. Thus, several strategies have been proposed to combat the drug resistance resulting from the poor accumulation of drugs in cancer cells. These include the modulation of drug influx transporters’ expression and function in cancer cells as well as the utilization of highly expressed transporters by targeting them with prodrugs and nanoparticles.

The present review aims to summarize the information about SLC transporter expression in different cancers compared to healthy tissues and the current research efforts to identify the role of low SLC drug transporter expression in anticancer drug resistance. In addition, recent advances in SLC transporter-targeting strategies, including the development of transporter-utilizing prodrugs, nanocarriers and the modulation of SLC transporter expression in cancer cells, are discussed.

## 2. Anticancer Drug Resistance

Multidrug resistance has been recognized as a major factor limiting the effectiveness of anticancer therapy [5]. Multidrug resistances are thought to cause treatment failure in more than 90% of patients with metastatic cancer [12]. After Juliano and Ling (1976) demonstrated that P-glycoprotein (P-gp) correlated with the degree of drug resistance in Chinese hamster ovary cells, the phenomenon of tumor resistance to anticancer drugs has received considerable attention and become a hotspot in cancer research [13]. Multidrug resistance to anticancer drugs has been defined as the cross-resistance or non-responsiveness of cancer cells to the cytostatic or cytotoxic actions of different drugs with various structural or functional properties and different molecular targets [5]. There are two main factors responsible for multidrug resistance [14]. The first is individual specificity in terms of pharmacokinetic processes such as the absorption, distribution, metabolism and clearance of the drug, which influence the delivery of drug to tumor site. This factor is defined by the genetic pattern of the patient. The second factor is tumor-specific and is also called the pharmacodynamic factor. It is dependent on the tumor’s origin, vasculature and tissue function.

The use of state-of-the-art -omics and functional techniques has resulted in a considerable increase in our understanding of drug resistance and the ability to identify the genes and signaling pathways involved in the response of tumors to a certain drug treatment. In addition, the molecular signatures and genotypes that predict the effects of anticancer drugs are better understood. This information can aid in the identification of novel therapeutic targets and approaches for overcoming drug resistance. Advances in the understanding of the molecular biology of cancer have shifted anticancer drug development from cytotoxic drugs towards agents targeting specific molecular changes in tumors. However, a diverse range of resistance mechanisms has limited the success of targeted drugs leading to various patient responses. Interestingly, the cytotoxic and targeted anticancer drugs share similar mechanisms of drug resistance. Therefore, information about the mechanisms of resistance to cytotoxic drugs can be translated to elucidate the mechanisms of resistance to novel targeted agents.

## 3. Mechanisms of Anticancer Drug Resistance

**Figure 1:**
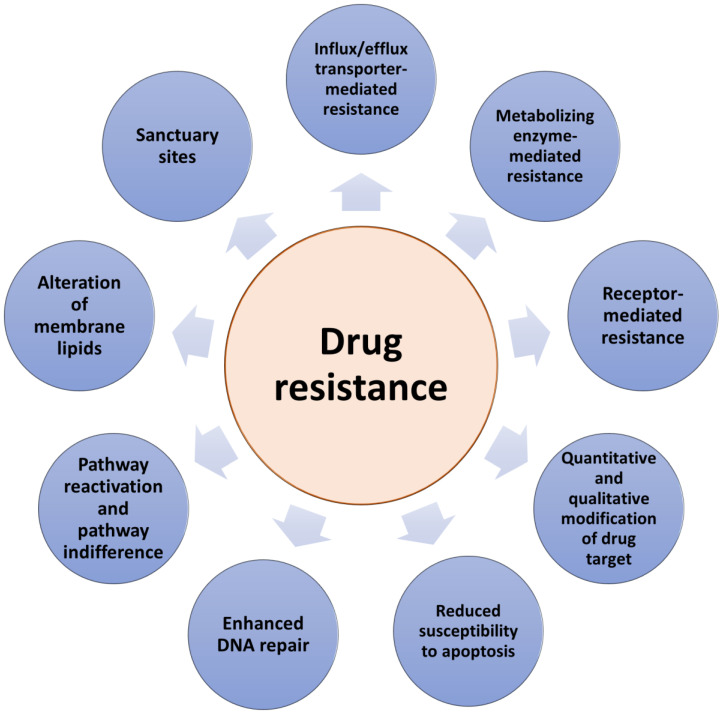
The main mechanisms of anticancer drug resistance.

There are two types of resistance to anticancer therapeutics: intrinsic and acquired. Intrinsic resistance to drug therapy occurs in tumors already before starting the treatment making the therapy ineffective. In contrast, acquired drug resistance can be developed during treatment by tumors that were initially sensitive to drug action. Previously, intrinsic and acquired resistance have been considered separately possessing binary differences. However, in practice, tumors develop resistances that include combinations of both types [15]. Drug resistance can result from a range of various molecular mechanisms (Figure 1). The colony of proliferating cancer cells may be located in sanctuary sites such as, for example, the central nervous system, protected by the blood–brain barrier. Sanctuary sites limit the delivery of anticancer drugs and the possibility of reaching therapeutic drug concentrations at the target site [15]. Other mechanisms include drug target mutations arising due to drug administration, the loss of a cell surface receptor, an increased extent of drug efflux and/or a decreased extent of drug influx, alterations in drug metabolism, or alterations in membrane lipids [16]. In addition, due to their highly adaptable nature, tumors can develop various other molecular responses, such as the up-regulation of the therapeutic target expression and/or the activation of alternative survival signaling pathways and the inactivation of downstream death signaling pathways, all of which can lead to drug resistance [14,17,18]. Moreover, epigenetic changes and the impact of the local tumor microenvironment can also contribute to anticancer drug resistance [19,20]. One of the main obstacles to overcoming acquired resistance is the ability of tumors not only to develop resistance to the drugs initially used but also to become cross-resistant to other drugs with different mechanisms of action. Moreover, due to the high degree of molecular heterogeneity of tumors [21], drug resistance can be caused by the therapy-induced selection of a resistant minor subpopulation of the cells presented in the original tumor. Importantly, it is necessary to elucidate the specific type of drug resistance in cancer patients in order to effectively combat it. Therefore, advances in determining the mechanisms by which cancer cells elude treatment are a key factor in the development of new strategies to overcome anticancer drug resistance and have a significant impact on the survival of cancer patients.

## 4. Transporter-Mediated Anticancer Drug Resistance

**Figure 2:**
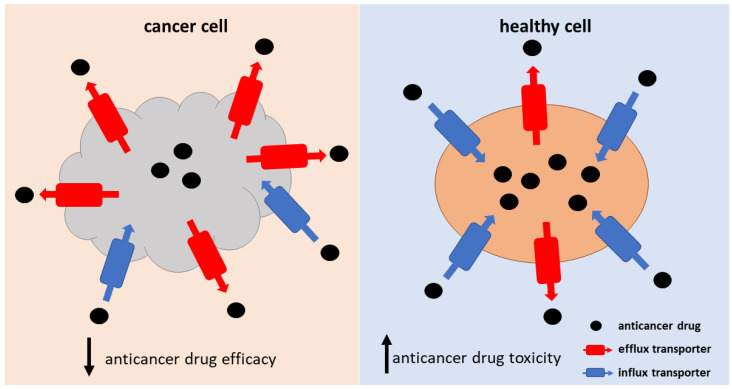
Schematic representation of the consequences of transporter-mediated resistance to anticancer drugs in cancer and normal cells.

Transporter-mediated drug resistance plays a crucial role in the process of anticancer drug accumulation into the cancer cells, affecting the drug concentrations at the target site. Insufficient anticancer drug delivery into the cancer cells can result from the up-regulation of efflux transporters and/or the down-regulation of influx transporters expressed at the membrane of the cancer cells (Figure 2). In addition, altered transporter activity on cell organelle membranes can affect the intracellular sequestration of drugs, leading to drug resistance. The altered transporter activity can be a result of changes in protein expression, localization, post-translational modifications (PTM), or mutations. Furthermore, the pH of the tumor microenvironment can affect the drug’s affinity to the transporters [22]. One might assume that one of the strategies for overcoming transporter-mediated drug resistance would be an increase in dosage, which would improve the poor accumulation of the drug to the cancer cells. However, as drug transporters are expressed also in healthy tissues, this approach can result in increased drug toxicity (Figure 2). Therefore, alternative ways to overcome transporter-mediated drug resistance should be found. In this review, we summarize the information about the potential strategies for overcoming transporter-mediated drug resistance and critically evaluate their applicability in clinical practice in the corresponding chapter (Section 6.). Another obstacle in solving the issue of transporter-mediated drug resistance is the fact that anticancer drugs are often substrates of several efflux and influx transporters. Moreover, for many anticancer drugs, the transporters responsible for their influx and efflux across cell membranes have not been identified, or the findings are inconclusive or contradictory (Table 1). This hinders the process of revealing the specific mechanisms of drug resistance and, consequently, the development of strategies aimed at restoring drug efficacy.

The most studied mechanism of transporter-mediated drug resistance is the increased efflux of drugs from cancer cells via adenosine triphosphate (ATP)-binding cassette (ABC) transporters. The ABC transporter superfamily consists of 48 human ABC genes [23]. ABC transporters utilize the energy of the hydrolytic reaction of ATP to efflux solutes from the cell or into the organelles against their concentration gradient [24,25]. The increased expression and function of ABC transporters in cancer cells has been shown to be associated with the decreased cellular accumulation of anticancer drugs and the MDR phenotype in cancers [26]. The most extensively characterized ABC transporters involved in MDR are ABCB1 (P-gp), ABCC1 (multidrug resistance-associated protein 1, MRP1) and ABCG2 (breast cancer resistance protein, BCRP). Since the discovery of ABCB1 and ABCC1-mediated MDR, several inhibitors of these and other ABC transporters have been developed (e.g., valspodar, elacridar, tariquidar and zosuquidar) but have demonstrated unsatisfactory results in clinical trials. However, the strategy to reverse anticancer drug resistance via the inhibition of ABC transporters remains a high priority and a focus of cancer research. Information about the role of ABC transporters in MDR can be found in recent extensive reviews [8,26,27], while the focus of this article is on the role of SLC transporters in anticancer drug resistance.

**Table 1:** SLC transporters involved in anticancer drug delivery, their expression in normal tissues and cancers.

Gene Name	Protein Name	Natural Substrates	Anticancer Drug Substrates	Tissue Expression	Expression in Cancer Compared to Normal Tissues *	References
SLC2A2	GLUT2	Glucose, glucosamine	Streptozotocin	Liver, pancreatic beta-cells, intestinal and renal epithelial cells	High expression: hepatocellular carcinoma ^c^, invasive ductal carcinoma ^c^, invasive colon tubular carcinoma ^c^, pancreatic adenocarcinoma ^c^, lung mesothelioma ^c^,	[28,29,30,31,32]
SLC7A5	LAT1	Phenylalanine, leucine, tryptophan	Melphalan, acivicin	Brain (endothelial cells), testis, retina, esophagus, testis, placenta and bone marrow	High expression: colorectal cancer ^a^, gliomablastoma ^b^, triple-negative breast cancer and HER2-positive breast cancers and MYC driver ER-positive breast cancer ^c,d^	[33,34,35,36,37,38,39,40,41,42,43]
SLC19A1	RFC1	Reduced folates, antifolates	Methotrexate, pemetrexed	Ubiquitous	High expression: non-small cell lung carcinoma and squamous cell carcinoma ^c^, MYCN- amplified neuroblastoma, colorectal carcinoma ^d^, urothelial bladder carcinomas ^d^ Low expression: ovarian cancers ^c^	[44,45,46,47,48,49,50,51,52,53]
SLCO1A2	OATP1A2	Bile salts, organic anions and cations	Imatinib, methotrexate	Brain (endothelial cells), kidney, intestine, liver, eye	High expression: breast cancer, glioblastoma ^c^ Low expression: colorectal carcinoma liver metastases ^a^, colorectal carcinoma ^d^,	[35,54,55,56,57,58,59]
SLCO1B1	OATP1B1	Bile salts, organic anions	Cisplatin, carboplatin, Oxaliplatin, regorafenib, belzutifan, SN-38, etoposide, tamoxifen, sorafenib	Liver	High expression: ovarian ^d^, colon ^d^ and pancreatic^c^ cancers, castration resistant prostate cancer metastases ^d^ Low expression: hepatocellular carcinoma ^a^, low in colorectal carcinoma liver metastases ^a^,	[35,54,55,60,61,62,63]
SLCO1B3	OATP1B3	Bile salts, organic anions	Nilotinib, belzutifan, docetaxel, SN-38, oxaliplatin, carboplatin, cisplatin, imatinib, gefitinib, sorafenib, belzutifan	Liver	High expression: breast cancer ^c,d^, colorectal carcinoma ^c,d^, castration resistant prostate cancer metastases ^d^ Low expression: hepatocellular carcinoma^a^, colorectal carcinoma liver metastases ^a^	[35,55,60,63]
SLCO2B1	OATP2B1	E-3-S, DHEAS	Etoposide, erlotinib	Liver, placenta, intestine, eye	High expression: prostate cancer with high Gleason score ^d^ and castration resistant prostate cancer metastases ^d^ Low expression: hepatocellular carcinoma ^a^, low in colorectal carcinoma liver metastases ^a^	[35,63,64]
SLCO4C1	OATP4C1	L-homoarginine	Methotrexate	Kidney	High expression: castration resistant prostate cancer metastases ^d^	[35,63]
SLC22A1	OCT1	Organic cations	Dasatanib, nintendanib	Liver, small intestine, kidney, lung, heart, skeletal muscle, brain (endothelial cells, neurons), adipose tissue, immune cells	Low expression: hepatocellular carcinoma ^a^, colorectal carcinoma liver metastases ^a^, cholangiocellular carcinoma ^c,d^	[60,65,66,67,68,69,70]
SLC22A2	OCT2	Organic cations	Cisplatin, oxaliplatin	Kidney, small intestine, trachea and bronchi placenta, thymus, brain (neurons, endothelial cells), inner ear	High expression: clear cell renal carcinoma ^c,d^ Low expression: hepatocellular carcinoma ^c^	[65,67,68,70,71,72,73,74,75]
SLC22A3	OCT3	Organic cations	Oxaliplatin	Heart, skeletal muscle, brain (neurons, glial cells, choroid plexus), small intestine, liver, lung, kidney, urinary bladder, mammary gland, skin blood vessels	High expression: head and neck squamous cell carcinoma ^c^, colorectal carcinoma ^c^ Low expression: colorectal carcinoma liver metastases ^a^, hepatocellular carcinoma ^c^, cholangiocellular carcinoma ^c,d^	[55,65,68,69,76,77,78,79,80,81,82,83,84]
SLC22A4	OCTN1	Ergothioneine, zwitterions, organic cations	Doxorubicin, mitoxantrone, imatinib, cytarabine	Kidney, intestine, spleen, heart, skeletal muscle, brain, mammary gland, thymus, prostate, airways, testis, eye, foetal liver, sperm, immune cells	Not found	[66,85,86,87,88,89,90]
SLC22A5	OCTN2	Zwitterions (L-carnitine), organic cations	Etoposide, imatinib	Skeletal muscle, kidney, prostate, lung, pancreas, heart, small intestine, adrenal gland, thyroid gland, liver	High expression: ER-positive breast cancers ^d^, glioma	[66,91,92,93,94,95,96]
SLC22A6	OAT1	Organic anions	Methotrexate, bleomycin	Kidney, placenta, choroid plexus	Low expression: kidney renal clear cell carcinoma and kidney renal papillary cell carcinoma ^d^	[97,98,99,100,101,102]
SLC22A7	OAT2	Organic anions	Irinotecan, docetaxel, 5-fluorouracil	Liver, kidney, eye	Low expression: colorectal carcinoma liver metastases ^a^, kidney renal clear cell carcinoma and kidney renal papillary cell carcinoma ^d^	[55,97,102,103,104,105,106,107]
SLC22A8	OAT3	Organic anions	Methotrexate	Kidney, choroid plexus	Low expression: kidney renal clear cell carcinoma and kidney renal papillary cell carcinoma ^d^	[97,98,99,100,101,102]
SLC28A1	CNT1	Pyrimidine nucleosides and adenosine	Gemcitabine	Kidney, liver, small intestine, bone marrow macrophages and the brain	High expression: serous, mucinous and endometroid ovarian carcinomas ^c^, uterine cervix carcinomas ^c^ Low expression: clear cell ovarian carcinomas ^c^, pancreatic ductal adenocarcinoma ^d^	[108,109,110,111,112,113,114,115]
SLC28A2	CNT2	Purine nucleosides and uridine	Gemcitabine,5-fluorouridine, 5-fluoro-2′-deoxyuridine	Heart, skeletal muscle, liver, kidney, intestine, pancreas, placenta, brain, spleen, rectum, colon, immune system	High expression: lung, ovary, uterus and prostate cancers ^d^ Low expression: hepatocellular carcinoma, colorectal carcinoma, colorectal carcinoma liver metastases ^d^, kidney, stomach, rectum and small intestine cancers ^d^	[110,113,116,117,118,119,120,121]
SLC29A1	ENT1	Nucleosides, nucleobases, creatinine, guanidine, thiamine	Gemcitabine, cytarabine, 5-fluorouracil, 6-mercaptopurine	Ubiquitous	High expression: ovarian, endometrial and uterine cervix carcinomas ^c^ Low expression: pancreatic ductal adenocarcinoma ^d^, prostate cancer ^d^	[35,113,114,115,122,123,124,125]
SLC29A2	ENT2	Nucleosides, creatinine, thiamine, carnitine	5-fluorouracil, gemcitabine	Ubiquitous	High expression: mantle-cell lymphoma ^d^, hepatocellular carcinoma ^d^, ovarian, endometrial and uterine cervix carcinomas ^c^	[35,113,114,122,123,124,125,126,127]
SLC31A1	CTR1	Copper (I)	Cisplatin, carboplatin, oxaliplatin	Liver, lung	High expression: bladder cancer ^c^	[128,129,130,131,132]
SLC46A1	PCFT	Reduced folates, folic acid	Pemetrexed	Small intestine, choroid plexus, kidney, liver, placenta, retinal pigment epithelium	High expression: colorectal cancer ^d^, ER-positive breast cancer ^d^	[51,133,134,135,136,137,138,139,140,141]
SLC47A1	MATE1	TEA, MPP	Oxaliplatin	Liver, kidney, muscle	Low expression: KRAS-driven colorectal carcinoma ^c^	[142,143,144,145,146]
SLC47A2	MATE2	TEA, MPP	Oxaliplatin	Kidney	Not found	[143,146,147,148]

* The information includes only reports in which transporter expression was compared between healthy and cancer tissue and differences were detected, ^a^ protein expression measured by quantitative targeted absolute proteomics, ^b^ protein expression measured by western blot, ^c^ semiquantitative immunohistochemistry, ^d^ mRNA expression.

## 5. SLC Transporters in Cancer

The SLC transporter superfamily represents another group of transmembrane transporters that play important role in anticancer drug resistance by mediating the influx and efflux of solutes across the plasma and intracellular membranes [23]. The first SLC nomenclature system was presented by Matthias A. Hediger and Phyllis McAlpine in the 1990s. Currently, the superfamily consists of over 60 families including more than 400 genes, with new transporter genes continuously being discovered (http://slc.bioparadigms.org/, (accessed on 14 November 2022)). The SLC transporters, which include facilitative and secondary active transporters, are responsible for the passage of essential nutrients and energy metabolites, e.g., glucose, amino acids, monocarboxylic acid, oligopeptides, nucleosides and water-soluble vitamins [149]. In addition, SLCs mediate the cellular uptake of drugs including anticancer agents (Table 1). However, for many anticancer drugs (Table 1), the knowledge about the transporters responsible for their cellular uptake is limited. These include cyclin and cyclin-dependent kinase inhibitors, poly adenosine diphosphate-ribose polymerase inhibitors, Kirsten rat sarcoma virus inhibitors and several tyrosine kinase inhibitors. In addition, for some of the drugs, such as sorafenib, the available data on the transporters responsible for their cellular uptake are contradictory [150,151]. As cancer cells require a high energy and nutrition content, several nutrient transporters were shown to be up-regulated in cancer cells [152,153]. In contrast, the drug-transporting SLCs, which are not necessary for cancer cell proliferation and survival, have been shown to be down-regulated, leading to a reduced accumulation of drugs in the cancer cells (Table 1).

Reliable methods are necessary for the correct interpretation of the mechanism of drug resistance and for understanding whether low SLC-transporter-mediated drug delivery plays a role in it. The accurate knowledge of the resistance mechanism facilitates the correct selection of a drug using a different transporter for cell uptake, or the selection of a strategy to increase anticancer drug uptake. In addition, better knowledge of the expression and function of SLC drug transporters in cancers can aid in the design of anticancer drugs and the development of delivery strategies in order to avoid the occurrence of intrinsic or acquired drug resistance due to a low SLC transporter expression. Currently, the estimation of drug delivery to tumors is difficult due to the lack of quantitative information about transporter protein expression in different cancer types. When culturing cancer cell lines, there is a selection of the fastest growing clones, and the cells can undergo genetic and epigenetic changes [154]. These changes can also affect the SLC transporter expression, thus hampering the ability of in vitro systems to reproduce the tumor transporter expression in patients. In addition, it is difficult to reproduce the heterogeneity of tumors and the tumor microenvironment, which has complex effects on the transporter expression, in in vitro cancer cell culture systems [154]. Therefore, in vitro drug cell accumulation experiments may not be good predictors of drug delivery to tumors. The mRNA expression analysis of tumors, although very useful, may not always correlate with the protein expression of transporters in the tumors. Therefore, predictions of drug exposure based only on mRNA expression may be misleading. Furthermore, confirmation of the drug transporter localization on the plasma membrane is of great importance. For example, the cancer-type OATP1B3 transporter has a high expression in colorectal carcinomas, but its expression is mainly detected in lysosomes, and, thus, cannot facilitate the cell uptake of its substrates [155,156]. Similarly, knowledge of the PTMs of transporters in cancer cells is crucial due to their effect on the transporter function [157]. Importantly, there is a lack of data about the drug transporter expression in different cancer subtypes, and the effect of the different oncogenes driving the cancers, on drug transporter expression. This information would allow the development more efficient drug delivery strategies for targeting therapies against specific subtypes of cancers. In the next chapter, we summarize the current knowledge of the main drug transporting SLCs and their expression in cancers versus in normal tissues and review the means of overcoming anticancer drug resistance due to a low SLC drug transporter expression.

### 5.1. Drug Transporters

#### 5.1.1. Glucose Transporter 2

The facilitative sugar transporters of the SLC2A family (GLUT) mediate the sodium-independent passage of glucose across the cell membrane [29]. Among the 14 members of the GLUT family, only GLUT2, encoded by SLC2A2, is known to play a role in the delivery of anticancer drugs. However, the number of known drugs transported by GLUT2 is limited to the antineoplastic agent streptozotocin, used for the treatment of neuroendocrine tumors [158]. GLUT2 possesses a high affinity for glucosamine and a low-affinity transporter for glucose, galactose, mannose and fructose. GLUT2 is highly expressed in hepatocytes, pancreatic beta-cells and intestinal and renal epithelial cells [159,160,161]. In the liver, it is responsible for the uptake of glucose by the hepatocytes for glycolysis and glycogenesis, as well as the efflux of glucose from the hepatocytes into the circulation during gluconeogenesis [28]. In enterocytes, GLUT2 is localized on the basolateral membranes and regulates the glucose efflux from cells into the circulation. In the kidneys, it facilitates the reabsorption of glucose from the glomerular filtrate, while, in the pancreatic beta-cells, GLUT2 acts as a glucose sensor controlling the uptake of glucose by beta-cells [28]. GLUT2 is a low-affinity, high-capacity transporter [162]. In hepatocellular carcinoma, the SLC2A2 mRNA and GLUT2 protein expressions were found to be higher than those of other GLUTs and were associated with poor overall patient survival [30,31]. Moreover, an immunohistochemical analysis performed by Godoy et al. (2006) revealed a high expression of GLUT2 in invasive ductal carcinoma, invasive colon tubular carcinoma, pancreatic adenocarcinoma and lung mesothelioma [32].

#### 5.1.2. Large Neutral Amino Acids Transporter Small Subunit 1

Large neutral amino acids transporter small subunit 1 (LAT1, encoded by SLC7A5) mediates a Na^+^–and pH-independent exchange of large branched-chain and aromatic neutral amino acids such as phenylalanine, leucine, isoleucine, tryptophan, histidine and tyrosine in antiport with histidine, tyrosine and glutamine with a 1:1 stoichiometry [33,34]. The transporter LAT1 is covalently linked (via a disulphide bond) with the heavy chain subunit (known as CD98 or 4F2hc, SLC3A2), a glycoprotein acting as a molecular chaperone localizing LAT1 at the plasma membrane [163]. The high mRNA expression of SLC7A5 was found in human tissues such as the cerebral cortex, retina, esophagus, testis, placenta and bone marrow [35]. Moreover, a higher expression of LAT1/SLC7A5 in tumors compared to normal tissue was confirmed for colorectal cancer in absolute protein level [36,37], glioblastoma in protein level [38], triple-negative and human epidermal growth factor receptor 2 (HER2)-positive breast cancers, as well as for MYC driver estrogen receptor (ER)-positive breast cancer, in the mRNA and protein levels [39]. Several studies revealed an association between a high expression of LAT1 and a significantly shorter survival in many types of cancer, indicating that this transporter may serve as a prognostic biomarker to predict the outcome in different cancer types [40,41]. In addition, the [^18^F] or [^11^C] labeling of the LAT1 substrate has been used for cancer diagnosis by PET imaging and is discussed in our recent review [164].

LAT1 has been an attractive target for the cancer delivery of drugs and prodrugs, as exemplified by the anticancer drugs melphalan and acivicin [42,43,165]. However, the high expression of the transporter in normal tissues, including the blood–brain barrier, makes the utilization of this transporter for targeted cancer delivery challenging. For example, acivicin has failed due to the unacceptable central nervous system toxicity caused by the high distribution of the compound to the brain.

#### 5.1.3. Reduced Folate Transporter

The reduced folate transporter, also known as reduced folate carrier 1 (RFC1), encoded by SLC19A1 refers to the SLC19 family of transporters responsible for the uptake of water-soluble vitamins into cells. RFC1 plays a major role in folate homeostasis. The transporter is a temperature- and pH-dependent and Na^+^-independent exchanger of folates with intracellular inorganic and organic anions [44,45,46]. In addition, the transporter is responsible for the uptake of antifolate chemotherapeutic agents, i.e., methotrexate and pemetrexed [47,48]. SLC19A1 single-nucleotide polymorphisms (SNPs) have shown to be associated with the altered transport of these anticancer drugs, resulting in altered therapeutic responses in individual patients [49]. In humans, this transporter is widely expressed in the body [46]. In addition, a high expression of the transporter was detected in non-small cell lung carcinoma and squamous cell carcinoma (on the protein level), as well as in MYCN-amplified neuroblastoma, colorectal carcinoma and urothelial bladder carcinomas (on the mRNA level) [50,51,52]. In contrast, low RFC1 protein expression was found in ovarian cancers [53].

#### 5.1.4. Organic Anion Transporting Polypeptides

The members of the organic anion transporting polypeptide (OATP) transporter superfamily are encoded by the SLCO genes and mediate a Na^+^- and ATP-independent cellular uptake of a wide range of structurally unrelated compounds [54]. In humans, the superfamily consists of 11 OATPs and is divided into six families (OATP1-6) based on a 40% amino acid sequence identity [166]. The protein structure of the OATPs is predicted to have 12 transmembrane domains with intracellular amino and carboxy termini. Generally, OATP substrates are large amphipathic organic anions with molecular weights greater than 300 Da and some cationic and neutral compounds.

OATPs transport a wide range of xenobiotics, including anticancer drugs as well as endogenous substrates such as prostaglandins, bile acids, thyroid hormones and steroid hormone conjugates [54]. According to the Human Protein Atlas, the mRNA/protein of OATPs are expressed in multiple tissues throughout the body, including those involved in the absorption, distribution and elimination of drugs, such as the intestinal (OATP1A2, OATP2A1, OATP1C1, OATP2B1, OATP3A1, OATP4A1, OATP4C1, OATP5A1), liver (OATP1A2, OATP1B1, OATP1B3, OATP1C1, OATP2A1, OATP2B1, OATP3A1, OATP4A1, OATP4C1, OATP5A1) and kidney (OATP1A2, OATP1C1, OATP2A1, OATP2B1, OATP3A1, OATO4A1, OATP4C1, OATP5A1) [35]. Therefore, the altered expression and/or function of the OATPs in these tissues may lead to changes in drug pharmacokinetics resulting in unexpected bioavailability and/or toxicities.

Among all of the OATPs, OATP1A2, OATP1B1, OATP1B3, OATP2B1 and OATP1C4 have been shown to play roles in mediating the delivery of anticancer drugs (Table 1) [66,167,168,169,170,171,172,173]. In addition to their presence in normal tissues, the expression of these transporters has been found to be up- or down-regulated in certain cancers (Table 1). Thus, OATP1A2 absolute protein expression has been found to be lower in liver cancer metastases compared to in histologically normal tissue [55]. Moreover, OATP1A2/SLCO1A2 and OATP1B3/SLCO1B3 mRNA and protein expression was significantly higher in malignant breast cancer tissue as compared to the surrounding non-malignant tissue [56,57]. SLCO1A2 mRNA expression was found in healthy colon tissue, while decreased levels were detected in polyps and in colon cancer tissue [58]. OATP1A2 protein expression was found to be significantly higher in glioblastoma tumor sections as compared to in non-neoplastic brain tissue [59]. Liver-specific OATP1B1 and OATP1B3 play important roles in the elimination of metabolites and xenobiotics from the body. However, liver cancers such as hepatocellular carcinoma and colorectal carcinoma liver metastases have an intrinsically low expression of these transporters [55,60], as they do not transport substrates, which are necessary for the survival of this type of cancer cell. This is a challenge in terms of drug delivery into liver tumors, as many drugs are substrates of OATP1B1 and/or OATP1B3. Therefore, in drug development for liver cancers, it should be considered that the drugs should be able to utilize other transporters for the cancer cell uptake. A higher expression of OATP1B1 was detected in ovarian and colon cancers and in pancreatic cancers (on the protein level) when compared to the normal corresponding tissues [58,61,62]. OATP1B3/SLCO1B3 protein and mRNA expression was higher in the colorectal carcinoma as compared to normal tissue, where it was not detected [155]. Moreover, mRNA expression levels of SLCO1B1, SLCO1B3, SLCO2B1 and SLCO4C1 were found to be higher in castration-resistant prostate cancer metastases as compared to in untreated prostate cancer [63]. Furthermore, SLCO2B1 mRNA expression was significantly higher in advanced prostate cancer with high a Gleason score [64].

#### 5.1.5. Organic Cation Transporters

The organic cation transporters 1–3 (OCT1-3) encoded by SLC22A1-3, respectively, are the members of SLC22 superfamily, which mediate the cellular transport of small (< 400 Da) cationic or neutral molecules [65]. The transport via OCTs is facilitative and Na^+^- and Cl^−^ -independent, occurring in both direction across the plasma membrane based on the electrochemical gradient of the transported substrates [74,93,174,175]. A variety of anticancer drugs have been identified as OCT transporter substrates, and several of them are listed in Table 1 [66,71,72,73,76]. OCT1/SLC22A1 is mainly expressed on the sinusoidal membrane of hepatocytes, and was detected in the small intestine, renal proximal tubular cells, the brain (neurons and endothelial cells of the blood–brain barrier), the heart, skeletal muscle, the lungs, adipose tissue and immune cells [67,68]. In contrast, OCT2/SLC22A2 is not expressed in the liver, while its expression was detected in the small intestine, placenta, skin, brain, kidney, trachea and bronchi and in the inner ear [67,68,93]. OCT3/SLC22A3 is widely expressed in human tissues including the kidney, liver, placenta, heart, skeletal, brain (neurons, glial cells and epithelial cells of the choroid plexus) and lungs [77,78,79,80,81].

In cancers, a low absolute protein expression of OCT1 was reported in hepatocellular carcinoma and colorectal carcinoma liver metastases [60]. In addition, a low OCT1/SLC22A1 protein and mRNA expression was detected in cholangiocellular carcinoma [69]. The OCT2/SLC22A2 protein and mRNA expression was found to be higher in clear cell renal carcinoma and lower in hepatocellular carcinoma as compared to non-cancerous tissues [70,75]. For OCT3, a high protein expression was detected in the head and neck squamous cell carcinoma and colorectal carcinoma [82,83]. In contrast, a low absolute protein expression of OCT3 was measured in colorectal carcinoma liver metastases [55]. In addition, a low OCT3/SLC22A3 expression was found in hepatocellular carcinoma (on protein level) and in cholangiocellular carcinoma (on the protein and mRNA levels) [69,84].

#### 5.1.6. Organic Cation Transporter Novel Type (OCTNs)

Organic Cation Transporters Novel Type (OCTNs) are other members of the SLC22 superfamily of membrane transporters, which are represented in humans by two transporters, OCTN1 and OCTN2, encoded by SLC22A4 and SLC22A5, respectively [65]. OCTN1 has 11 predicted transmembrane domains, while OCTN2 has 12 predicted transmembrane domains. OCTN1 can act as an organic cation/proton exchanger, a cation exchanger, or a Na^+^-dependent or Na^+^-independent zwitterion transporter. It mediates the transport of ergothioneine, the antioxidant amino acid and acetylcholine [85,86]. OCTN2 is a Na^+^-dependent, pH-sensitive high affinity co-transporter of L-carnitine [91]. In addition, OCTN2 can function as a polyspecific Na^+^-independent organic cation transporter, and mediates the transport of substrates in both directions across the plasma membrane [91]. Both transporters mediate the cellular uptake of anticancer drugs (Table 1) [66,87,92,176]. OCTN1 and OCTN2 are widely expressed in human tissues. Thus, the expression of OCTN1/SLC22A4 was detected in the kidney, colon, spleen, prostate, testis, heart, skeletal muscle, brain, lung, skin, thymus, bone marrow, cornea, blood-retina barrier, fetal liver, sperm and immune cells [88]. Drenberg et al. (2017) reported that OCTN1/SLC22A4 has variable expression in AML cells and that a high expression of it is a predictor of OCTN1 substrate treatment response [87]. Other studies on OCTN1 have shown that OCTN1 may be affected by the circadian rhythm, circulating testosterone levels and various cytokines [89,90]. OCTN2 expression was detected on the apical brush-border membrane of renal proximal renal tubules, the apical of small intestinal enterocytes, and in the heart, liver, skeletal muscle, etc. (Table 1) [93,94]. Moreover, a high expression of OCTN2/SLC22A5 was detected in ER-positive breast cancer (on mRNA levels) and glioblastoma (on mRNA and protein levels) [95,96].

#### 5.1.7. Organic Anion Transporters

Organic anion transporters (OATs) are additional polyspecific transporters that are the members of SLC22 superfamily. The human OATs include OAT1 (SLC22A6), OAT2 (SLC22A7) OAT3 (SLC22A8), OAT4 (SLC22A11), OAT5 (SLC22A10), OAT6 (SLC22A20), OAT7 (SLC22A9) and OAT10 (SLC22A13) [65]. These transporters have 12 predicted transmembrane domains composed of about 540–560 amino acids. OATPs are involved in the transport of a diverse range of low molecular weight substrates such as steroid hormone conjugates, biogenic amines, various drugs including anticancer agents and toxins [97]. Among all OATs, OAT1-3 has been shown to play a role in anticancer drug delivery (Table 1) [98,99,103,177,178]. In humans, OAT1 and OAT3 are kidney-specific transporters and are predominantly expressed in the basolateral membrane of proximal tubule cells [100,101]. In rats, Oat1 expression has been also found in the choroid plexus, skeletal muscle and placenta, whereas Oat3 was detected only in the choroid plexus [100,179,180,181]. OAT2 has a high expression in the liver, where it is thought to be localized to the sinusoidal membrane of hepatocytes [104,182]. In addition, it is expressed to lesser extent lower in the kidney, where it is localized in the basolateral membrane of proximal tubule cells in humans [105,106]. Moreover, OAT2 expression was also detected in the corneal epithelium [107]. SLC22A6-8 mRNA expression was found be lower in kidney renal cell carcinoma and kidney renal papillary cell carcinoma as compared to in normal tissues [102]. In addition, the absolute protein expression of OAT2 was lower in colorectal carcinoma liver metastases compared to in non-cancerous tissue [55].

#### 5.1.8. Concentrative Nucleoside Transporters

The SLC28 family consist of three concentrative nucleoside transporters (CNT1-3) encoded by SLC28A1-3, respectively [183]. The CNTs act as symporters which require inwardly directed Na^+^- or proton-dependent coupling. CNT1 is a Na^+^-dependent symporter for pyrimidine nucleosides, as well as nucleoside-based anticancer and other drugs with a stoichiometry of 1:1 (nucleoside: sodium) [108]. CNT2 is another Na^+^-dependent transporter mediating the passage of purine nucleosides, as well as of uridine and nucleoside-based anticancer and other drugs with a 1:1 stoichiometry of nucleoside: sodium transport [116,117]. CNT3 functions as a Na^+^-nucleoside or proton-nucleoside symporter [184]. All three transporters are 72-kDa proteins with a putative structure of 13 transmembrane domains [185]. Among the CNTs, CNT1 and CNT2 have been shown to mediate the transport of anticancer agents (Table 1) [109]. Both transporters are involved in gemcitabine cellular uptake [110]. CNT1 expression in pancreatic cancer cell lines correlated with a sensitivity to gemcitabine therapy [109]. Lang et al. (2001) demonstrated that CNT2 mediates the uptake of halogenated uridine analogues, such as 5-fluorouridine and 5-fluoro-2′-deoxyuridine, in hCNT2-transfected CEM-ARAC leukemia cells with resistance to cytarabine [116]. CNT1 is expressed mainly on the apical side of the epithelial and endothelial cells in different tissues, which include the liver, kidney, bone marrow macrophages, small intestine and brain [111,112,113]. Similarly, CNT2 is expressed in various tissues including the liver, kidney, spleen, heart, rectum, intestine, brain, placenta, pancreas, skeletal muscle, colon and immune system [113,118,119,120]. Moreover, an immunohistochemistry analysis revealed a high expression of CNT1 in serous, mucinous and endometroid ovarian carcinomas, serous and endometroid endometrial carcinomas and uterine cervix carcinomas [114]. A low protein expression of the transporter was found in clear cell ovarian carcinomas [114]. In addition, a low mRNA expression of SLC28A1 was detected in pancreatic ductal adenocarcinoma [115]. A high SLC28A2 mRNA expression was found in lung, ovary, uterus and prostate cancers, while a low expression was detected in hepatocellular carcinoma, colorectal carcinoma, colorectal carcinoma liver metastases, as well as in kidney, stomach, rectum and small intestine cancers [113,121].

#### 5.1.9. Equilibrative Nucleoside Transporters

Equilibrative nucleoside transporters (ENTs) transporters represent the SLC29 superfamily consisting of human ENT1-4 encoded by SLC29A1-4, respectively [183]. ENTs are considered as Na^+^-independent facilitative uniporters. However, the activity of the human ENT3 and ENT4 transporters has been shown to be stimulated at lower pH [186]. ENTs have 11 transmembrane domains and mediate the transport of nucleosides, nucleobases and nucleoside-derived therapeutics [122]. In terms of anticancer drug delivery, ENT1 and ENT2 play a role in the transport of chemotherapeutic agents, i.e., nucleoside analogues and nucleobases (Table 1) [123,124,125]. Both transporters are widely distributed throughout the body [35]. Moreover, the expression of ENT1 and ENT2 was found to be either up- or down-regulated in certain types of tumors compared to normal tissues. Thus, a high protein expression of both ENT1 and ENT2 was detected in ovarian, endometrial and uterine cervix carcinomas as measured by western blot analysis [114]. Moreover, SLC29A2 possessed a high mRNA expression in mantle-cell lymphoma and HCC, while a low mRNA expression of SLC29A1 was reported in pancreatic ductal adenocarcinoma and prostate cancer [113,115,126,127]. High ENT1 and ENT2 expression may influence the relative selectivity of nucleoside chemotherapy for malignant cells. Therefore, measurement of the expression of transporters may be used as a predictive tool for the evaluation of the effectiveness of the treatment in individual patients. For instance, a high ENT1 protein expression was associated with improved overall survival in patients administered gemcitabine in the ESPAC-3(v2) trial population [187]. Moreover, SLC29A1 mRNA expression significantly correlated with the gemcitabine resistance and IC50 values of 5-fluorouracil in vitro [188]. Hubeek et al. (2005) revealed a correlation between SLC29A1 mRNA expression and a sensitivity to cytarabine in childhood acute myeloid leukemia [189].

#### 5.1.10. Copper Transporter 1

Copper transporter 1 (CTR1), encoded by SLC31A1, is a protein consisting of 190 amino acids with three transmembrane domains, an extracellular N-terminal domain of approximately 67 and a C-terminal cytosolic tail of an approximately 15 amino acids [190,191]. CTR1 is the major influx transporter of copper in human cells. The copper transport via CTR1 is energy-independent, but potassium dependent, and results in transporter conformational changes [192,193]. Although CTR1 is widely expressed in the body, high levels of this transporter were detected in the liver and kidneys [128]. In addition, CTR1 protein expression was higher in the bladder tumor sections of patients as compared to the adjacent normal tissues, and it was found to correlate with the pathological outcome after platinum-based neoadjuvant chemotherapy in patients with muscle-invasive bladder cancer [129]. Importantly, CTR1 is a crucial mediator of the uptake of platinum-based anticancer drugs such as cisplatin, carboplatin, oxaliplatin [130,131,132]. The resistance to cisplatin due to decreased uptake is considered as a key limitation of cisplatin treatment, while CTR1 plays an important role in the development of resistance to cisplatin, leading to the ineffectiveness of the treatment of cancer [131,132]. Thus, reduced CTR1 expression might be associated with cisplatin resistance in patients, and the modulation of CTR1 expression in specific cancer cells can be a therapeutic strategy to overcome the transporter-mediated cisplatin resistance [194].

#### 5.1.11. Proton-Coupled Folate Transporter

Proton-Coupled Folate Transporter (PCFT), encoded by SLC46A1, is one of three transporters representing the SLC46A family, which has demonstrated its role in anticancer drug delivery of antifolates, such as pemetrexed [133,134,195]. At low pH, PCFT possesses a high affinity for both folic acid and the reduced folates [133]. The transporter is expressed in many tissues, with high levels at the apical brush-border membrane of the small intestine, the liver sinusoidal membrane, the apical membrane of the kidney, the choroid plexus, the placenta and the retinal pigment epithelium [135,136,137,138,139,140]. Moreover, a high mRNA expression of SLC46A1 was detected in colorectal carcinoma and ER-positive breast cancer [51,141].

#### 5.1.12. Multidrug and Toxin Extrusion Proteins 1 and 2

Multidrug and toxin extrusion proteins 1 and 2 (MATE1 and MATE2), encoded by SLC47A1 and SLC47A2, respectively, use the proton gradient for the transport of their substrates [196]. MATE1 and MATE2 are polyspecific antiporters which directly transport organic cations, such as tetraethylammonium (TEA) and 1-methyl-4-phenylpyridinium (MPP), into the urine and bile (only MATE2) [142]. MATE2 is an electroneutral, Na^+^-independent, pH-dependent proton antiporter which mediates the transport of organic cations and has two SLC47A2 splice variants with two protein products such as MATE2-K and MATE2-B [147,148,197]. The knowledge about the function, physiology and clinical importance of MATE2 has been mainly based on the information about MATE2-K. Both MATE1 and MATE2 have been shown to play a role in the uptake of the platinum-based antineoplastic drug oxaliplatin [143]. Thus, Fujita et al. (2018) demonstrated that oxaliplatin accumulated in Mate1-expressing cells, and Mate1 siRNA-injected rats possessed more severe neuropathy compared to the control animals [198]. MATE1 is predominantly expressed in the liver and kidneys with localization at the apical membranes of the bile canaliculi and renal tubules [144,145], while MATE2 expression was exclusively found in the apical membrane of proximal tubular cells [147,148]. Moreover, a low protein expression of MATE1 was found in KRAS-driven colorectal carcinoma [146].

## 6. Strategies to Overcome Low SLC Transporter Expression-Mediated Drug Resistance

To produce the pharmacological effect, anticancer drugs with intracellular target molecules should accumulate in cancer cells at a sufficient concentration. Importantly, the transporters predominantly responsible for anticancer drug cell uptake, such as members of the SLC22 and SLCO families, are often more highly expressed in healthy tissues than in cancer cells, leading to the unfavorable distribution of the anticancer drugs (Table 1). Furthermore, the acquired down-regulation of the SLC transporters responsible for the cellular uptake of anticancer drugs leads to the ineffectiveness of the treatment, and attempts to enhance the limited efficacy of the drugs by increasing their doses may lead to systemic toxicity. Therefore, several strategies aiming to increase uptake of drugs via SLC transporters in cancer cells have been proposed and investigated and are discussed in this chapter.

### 6.1. Modulation of Transporter Expression

**Figure 3:**
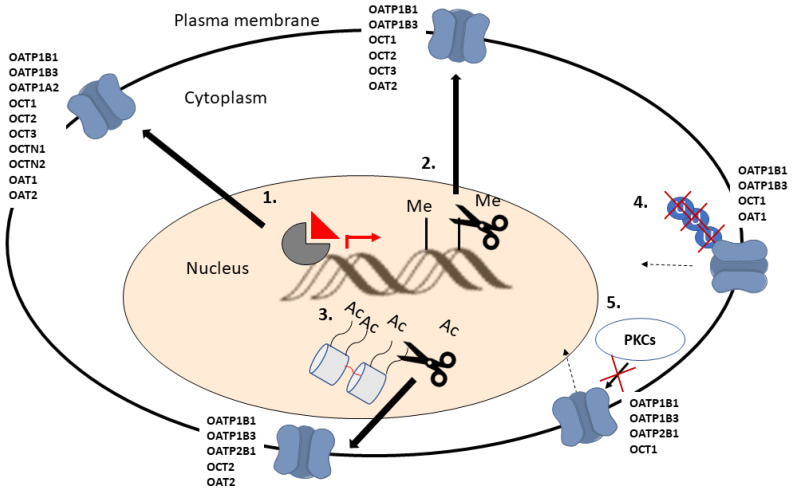
Schematic illustration of the molecular mechanisms which can be targeted to increase SLC drug transporter expression on cell plasma membrane. Nucleus receptor (1), DNA de-methylation (2) and histone de-acetylation (3) mediated increase in transporter expression. Ubiquitination inhibition mediated reduction of transporter recycling from the plasma membrane (4). Inhibition of Protein kinases C (PKCs) mediated internalization of transporters from plasma membrane (5).

Due to the expression of SLC transporters in normal tissues, the development of agents aimed to induce the expression of SLC transporters, which are down-regulated in cancer cells, can result in an increased accumulation of drugs in normal cells as well. For these reasons, identifying strategies for the tumor-selective modulation of SLC transporter expression and developing drugs targeted to these mechanisms is of great importance. Recently, Brouwer et al. (2022) and Zhou and Shu (2022) published extensive reviews on the transcriptional regulation of SLC transporters, providing an excellent overview of potential mechanisms to affect transporter expression in cancer cells [199,200]. Here, we describe the mechanisms affecting SLC drug transporter expression in cancer cells and the potential strategies for utilizing this knowledge for increasing drug accumulation to cancer cells (Figure 3). The described mechanisms also affect the expression of other SLC transporters, but the focus is on transporters known to take part in anticancer drug uptake.

#### 6.1.1. Impact of Nuclear Receptors on Transporter Expression

Nuclear receptors are a set of proteins that bind to specific regions of the DNA molecule that control gene expression by promoting or suppressing transcription, and are thus considered transcription factors [201]. The transcriptional regulation of the SLC drug transporter genes can be controlled by multiple nuclear receptors. Therefore, it is important to know the specific mechanism causing the low drug transporter expression in different cancer types to apply the correct means to increase the transporter expression. It should be noted that nuclear receptors also regulate efflux transporter expression [199], and this may abate the increased drug accumulation to cancer cells caused by the induced SLC transporter expression by nuclear receptor agonists.

Pregnane X receptor (PXR) is a transcription factor highly expressed in the liver and intestine, with several endogenous and exogenous ligands [202]. PXR is known to regulate the transcription of the SLC22A1, SLCO1B1, SLCO1B3, SLCO2B1 and SLCO1A2 transporters, which are responsible for the cell uptake of several anticancer drugs (Table 1) [200]. PXR affects the expression of SLCO1A2 in breast cancer cells [56]. However, as the transporter is highly expressed in this type of cancer the pharmacological activation of PXR would not likely increase drug efficacy (Table 1). PXR agonist rifampicin can increase the OATP1B1 expression and subsequent sorafenib cell accumulation and efficacy in a liver cancer cell line, HepG2 [203]. As the expression OATP1B1 is low in liver cancers, the PXR-mediated OATP1B1 expression induction may provide a means to increase the efficacy of OATP1B1 substrate anticancer drugs. However, at the moment, there is no clinical evidence to support the mentioned in vitro study. In addition, the possible induction of transporter expression in the liver by PXR agonists and the resulting changes in drug pharmacokinetics should be considered. Furthermore, as PXR regulates the expression of drug efflux transporters ABCG2, ABCB1 and ABCC2, the usability of the approach may be limited [199]. The role of PXR in regulating the SLC22A1 transporter is controversial, as different studies show either up- or down-regulation of the transporter upon PXR agonist binding [200].

Farnesoid X receptor (FXR) is a nuclear receptor highly expressed in the liver and intestine [204]. It controls the hepatic bile acid and triglyceride homeostasis by regulating bile acid synthesis, detoxification and transport. FXR is an interesting nuclear receptor in terms of anticancer drug disposition, as it regulates the expression of SLCO1B1, SLCO1B3 and SLC22A7 transporters [200]. FXR agonists can reduce tumor growth and metastasis in mouse models of liver and cervical cancers [205,206]. In addition, FXR agonists can reduce the cell proliferation and migration of breast, colon and liver cancer cells in vitro [207]. On the contrary, FXR agonists increased the migration and invasion of pancreatic cancer cells, and FXR inhibition reduced cell proliferation of lung cancer cells in vitro as well as tumor growth in vivo in mice [207]. Interestingly, FXR agonists can increase OATP1B1 and OATP1B3 mRNA and protein expression as well as activity in human liver cancer cells [199]. These data suggest that FXR agonists could be combined with OATP1B1 substrate anticancer drugs for the more efficient treatment of liver cancers and possibly breast and colon cancers. Importantly, FXR agonists have not been shown to increase the expression of drug efflux transporters, such as BCRP and P-gp, making it a more interesting approach to inducing anticancer drug accumulation to cancer cells than the use of PXR agonists. However, as FXR agonists affect the OATP1B1 transporter expression in healthy liver cells, also, the possible impact on OATP1B1 substrate anticancer drug pharmacokinetics may limit the usability of the approach.

Hepatocyte nuclear factors (HNFs) are transcription factors that regulate the transcription of a wide range of genes, including the SLC transporters SLCO1B1, SLCO1B3, SLCO2B1, SLC22A1, SLC22A6 and SLC22A7 [199,200,208]. The HNF-mediated OATP/SLCO transporter expression regulation can be complex. SLCO1B1 and SLCO1B3 expression is promoted by HNF1α and HNF4α, whereas SLCO2B1 expression is regulated by HNF4α. In addition, SLCO1B3 expression is down-regulated by HNF3β [199,208,209]. In hepatocellular carcinoma, the elevated expression of HNF3β represses SLCO1B3 expression [210]. Thus, the inhibition of HNF3β may provide means to increase SLCO1B3 expression and increase drug therapy efficacy in hepatocellular carcinoma.

Liver X receptor alpha (LXRα) is a nuclear receptor highly expressed in the liver but also expressed at a lower abundance in the kidneys, intestines, macrophages, lung, spleen and fat tissue [211]. LXRα regulates the cholesterol, fatty acid and glucose homeostasis [212]. In addition, LXRα regulates the transcriptional expression of the SLCO1B1, SLC22A6 and SLC22A7 transporters [200]. The LXRα agonists TO-901317 and GW3965 have been reported to increase the mRNA expression of SLCO1B1 in Huh7 hepatocellular carcinoma cells [213]. Moreover, LXRα activation can potentiate sorafenib efficacy in hepatocellular carcinoma cells and in patient-derived hepatocellular carcinoma tumor-bearing mice [214]. The sorafenib sensitizing effect was attributed to microRNA-378a transcription, and the potential role of LXRα activation-mediated elevated OATP1B1 expression and sorafenib cell accumulation was not investigated. However, the results show that the LXRα activation approach to increasing SLCO1B1 expression and anticancer drug cell accumulation may have potential.

Retinoid X receptor (RXR) forms heterodimers with several other nuclear receptors [215] which control the expression of several targets, including the SLC transporters SLCO1B1, SLCO2B1, SLC22A1 and SLC22A4 [200]. There are three types of RXR dimers: RXR homodimer, permissive heterodimers (PPAR/RXR, PXR/RXR, FXR/RXR) and non-permissive heterodimers (RAR/RXR, VDR/RXR and TR/RXR) [215]. RXR homodimer and permissive heterodimers are activated by RXR agonists, whereas the non-permissive heterodimers are activated only by the agonist of the partner receptor [215]. Austin et al. (2014) showed that, in CML cells, SLC22A1 expression can be induced by both permissive heterodimer PXR/RXR and PPAR/RXR and non-permissive heterodimer RAR/RXR [216]. However, it was not evaluated whether the elevated SLC22A1 transcription led to the increased intracellular accumulation and efficacy of OCT1/SLC22A1 substrate drugs.

Peroxisome-proliferator-activated receptors (PPARs) are nuclear hormone receptors including PPARα, PPARδ and PPARγ. PPARs take part in the regulation of cancer cell proliferation, survival, apoptosis and tumor growth [217]. Ligand binding and activation of PPARs heterodimerize with RXRs and regulate the expression of SLC22A1, SLC22A2 and SLC22A5 [200]. In a study by Wang et al. (2012), PPAR-α agonists significantly enhanced the anti-leukemic effects of imatinib in KCL22 cells and CD34+ primary cells through up-regulating the SLC22A11 gene expression and increasing the uptake of imatinib by CML cells [218]. Therefore, PPAR-α agonists could be potentially used in combination with imatinib and other OCT1/SLC22A1 substrates for combatting CML.

The retinoic acid receptor (RAR) is a nuclear receptor which can be activated by all-trans retinoic acid and 9-cis retinoic acid [219]. RAR heterodimerizes with RXR and binds to retinoic acid response elements complexed with a corepressor protein [220]. The binding of an RAR agonist leads to dissociation from the corepressor protein and the recruitment of a coactivator protein promoting the transcription of target genes, including SLCO1B1, SLCO2B1, SLCO1A2 and SLC22A1 [200]. In hepatoma, HepaRG cells and human hepatocytes, the SLCO1B1, SLCO2B1, SLC22A1 and SLC22A7 expression was down-regulated by an RAR agonist, all-trans retinoic acid [221]. However, the expression of SLCO1B3 was differently regulated by the RAR agonist, as the expression in the hepatoma cells was increased, whereas, in the hepatocytes, the expression was decreased.

The aryl hydrocarbon receptor (AhR) can regulate the transcription of SLCO1B1, SLCO1B3, SLCO2B1, SLC22A6 and SLC22A7 transporter gene expression [200]. The AhR ligand shikonin has been reported to effectively up-regulate the transcription of Slco transporters through the activation of AhR in primary rat hepatocytes [222]. However, in human primary hepatocytes, an AhR agonist, 2,3,7,8-tetrachlorodibenzo-p-dioxin, represses the mRNA expression SLCO2B1 [223]. AhR agonists and antagonists have been investigated as potential anticancer drugs [224]. It has been shown that AhR agonists can decrease breast cancer proliferation and migration [225]. However, the use of AhR agonists as anticancer agents against breast cancer may lead to the down-regulation of SLCOs, and, therefore, the combination with OATP/SLCO substrate drugs should be investigated thoroughly. On the other hand, oral squamous cell carcinoma has been reported to be sensitive to AhR antagonists [226]. The use of AhR antagonists may sensitize these cancer cells to OATP and OAT substrate anticancer drugs, and the investigation of such a drug combination can be merited. However, it should be kept in mind that AhR also induces the expression of ABC export proteins, thus potentially decreasing the intracellular concentration of anticancer drugs [227,228].

The constitutive androgen receptor (CAR) is a nuclear receptor that regulates the transcription of genes involved in xenobiotic metabolism [229]. CAR is mainly expressed in the liver and the intestine [230]. CAR activation with phenobarbital has been reported to decrease the gene expression of SLC22A1, SLCO2B1 and SLC22A7 in primary human hepatocytes [231]. Interestingly, CAR agonists can also increase the protein expression of efflux transporters P-gp, MRP2 and BCRP [231]. Therefore, it would be tempting to investigate the effects of CAR antagonists on transporter expression in cancer cells with a low abundancy of SLC22A1 and SLCO2B1.

#### 6.1.2. Impact of Epigenetics on Transporter Expression

Epigenetic events including DNA methylation in cytosine-phosphate-guanine (CpG) islands in the promoter region of a specific gene and histone modification can lead to the impaired expression or silencing of SLC transporter genes in cancer cells. For instance, OCT1 protein expression was significantly down-regulated in hepatocellular carcinoma samples compared to the normal adjacent liver tissue and was associated with DNA methylation [70]. In addition, the mRNA expression of SLCO1B3 (OATP1B3) in cancer cells as well as in normal tissue was shown to be associated with the DNA methylation status around the transcriptional start site. Thus, in two SLCO1B3-positive cell lines, such as colorectal carcinoma DLD-1 and bile duct carcinoma TFK-1 cells, CpG dinucleotides around the transcriptional start site were significantly hypomethylated. In contrast, in two SLCO1B3-negative cell lines, i.e., hepatocellular carcinoma HepG2 and colorectal adenocarcinoma cells, Caco-2 cells, and, in the kidney, CpG dinucleotides were hypermethylated [232]. The treatment with an inhibitor of DNA methyltransferase significantly induced SLCO1B3 mRNA expression in both the HepG2 and Caco-2 cell lines. Later, Imai et al. (2013) further demonstrated that the mRNA expression of a cancer-type variant of SLCO1B3 in cancer cell lines is regulated by DNA methylation-dependent gene silencing involving methyl-DNA binding protein 2 (MBD2) [233]. In this study, the treatment with a DNA methyltransferase inhibitor and the siRNA knockdown of MBD2 significantly induced the mRNA expression of methyl-DNA binding proteins in HepG2 and Caco-2 cells. Thus, the development of agents targeting epigenetic events such as DNA methylation may provide attractive opportunities for increasing the expression of the down-regulated SLCs and enhancing the uptake of anticancer drugs to the cancer cells.

PTMs of the DNA-binding histones is a gene expression regulation mechanism in cells [234]. The chemical modification of histones is mediated by histone acetylase, deacetylase, methyltransferase, and demethylase enzymes [235]. Histone modification regulates gene expression by affecting the binding between histones and DNA double strands and altering the conformation of nucleosomes, or by affecting the binding between the transcription factors and the promoter of a gene [236]. The expression of various genes are controlled by histone modification, including SLC drug transporters such as SLCO1B1, SLCO1B3, SLC22A2 and SLC22A7 [200]. It has been shown that OAT2 expression is regulated by histone acetylation in hepatocellular carcinoma [237]. Interestingly, the up-regulation of histone acetylation with suberoylanilide hydroxamic acid increased the OAT2 expression and enhanced fluorouracil efficacy in liver cancer cells [237]. In addition, Zhu et al. (2019) found that a histone deacetylase inhibitor, suberoylanilide hydroxamic acid, significantly induced the gene and protein expression of OCT2 in renal cell carcinoma cells, enhanced the cellular accumulation of oxaliplatin, and significantly reversed drug resistance [238]. These reports indicate that histone deacetylase inhibitors have promise in overcoming low SLC transporter expression-mediated drug resistance. However, more research is needed to confirm the clinical usefulness of this anticancer treatment strategy.

#### 6.1.3. Impact of Post-Translational Modifications on Transporter Expression, Localization and Function

PTMs modulate protein functional expression through a wide range of molecular mechanisms including the addition of a functional group (e.g., phosphorylation), a sugar chain (e.g., glycosylation), lipids (e.g., palmitoylation) or a small protein (e.g., ubiquitination) on solvent accessible amino acid residues. All these PMTs have been shown to affect the function of many SLC transporter proteins like OCT2 [208,239,240,241,242], OATs and OATPs. The mechanism of PTM depends on the amino acid sequence, the structural and chemical features of the protein surface, and the availability of the required protein machinery and precursors to enable the modification [243,244]. PMTs mainly arise in response to various cellular stresses or stimuli and may be reversed depending on the type of modification and the intended fate of the signaling event. Multiple types of PMTs can occur to the transporter protein at the plasma membrane, which can alter the functional activity, protein internalization and recycling [157]. Moreover, for membrane transporters, including SLCs, PTM events are further complicated due to lipid-protein interactions, both of which are involved in the internalization and recycling of the transporters located at the plasma membrane [157].

Alam et al. (2017) determined the ubiquitination of OATP1B1 and OATP1B3, one of the major mechanisms through which proteins are degraded intracellularly, and the apparent substrate-dependent inhibitory effect of proteosome inhibitor bortezomib, a drug used for multiple myeloma treatment, on OATP1B3-mediated transport [245]. In another study, treatment with proteasome inhibitors bortezomib and carfilzomib increased the cellular levels of the ubiquitinated OAT1 protein and augmented the functional OAT1 levels at the plasma membrane [246]. Proteasome inhibitor therapy is often administered for an extended time in combination with other anticancer drugs to suppress the disease’s progression. It is currently unknown whether the activity of the SLC transporters may be induced by long-term treatment with proteosome inhibitors in cancer patients. However, strategies targeting PTMs to induce the functional expression of the SLC transporters down-regulated in cancer cells could open new perspectives for overcoming low SLC transporter-mediated drug resistance.

Protein kinases C (PKCs) regulate both SLC and ABC drug transporter activity, localization and expression [247]. The activation of PKCs has been shown to decrease the OATP1B1 and OATP2B1 protein expression in human primary hepatocytes and cause increased internalization from the plasma membrane of transfected HEK293 cells (OATP1B1) and cancer cells lines (OATP2B1) [247]. In addition, PKCs activation reduces OATP1B3 activity and OATP1B3 and OCT1 protein expression in human primary hepatocytes. Interestingly, PKCs activation has been shown to increase the P-gp expression and activity in human cancer cells, whereas PKCs activation had no effect on BCRP expression in human primary hepatocytes [247]. Therefore, PKCs inhibitors show promise as promoters of drug influx transporter expression in cancers with low OATP1B1, OATP1B3, OATP2B1 and OCT1 plasma membrane expression. However, more research is required to determine how effective the PCKs inhibitors are in increasing the plasma membrane expression of these transporters in different cancers, as majority of the studies so far have been performed in primary hepatocytes.

#### 6.1.4. Impact of Anticancer Drugs on Transporter Expression and Function

Anticancer drugs can impact the SLC drug transporter expression and function in the target cells and thus have an impact on the cell accumulation of the drug itself or other drugs used in combination. The possible impact of drugs on transporters should be considered when selecting an anticancer drug therapy. In addition, more research should be conducted in this field to gain a better understanding on the possible pharmacokinetic interactions of anticancer drugs in different cancers. Recently, tyrosine-protein kinase LYN was discovered to regulate OATP1B1 activity by tyrosine phosphorylation [248]. In addition, the study showed that 29 out of 46 FDA approved tyrosine kinase inhibitors significantly inhibited the LYN kinase and thus prevented OATP1B1 phosphorylation and reduced the transporter activity. As many tyrosine kinase inhibitors can reduce OATP1B1 activity, caution should be used in combining drug treatments involving tyrosine kinase inhibitors and substrates of OATP1B1. In addition to OATP1B1, tyrosine kinase inhibitors have been reported to inhibit CNT and ENT activity [249]. The nucleotide transporter inhibition by tyrosine kinase inhibitors has been proposed to be the underlying reason for the failures in tyrosine kinase inhibitors and nucleoside combination therapies [249]. In another study, it was shown that anthracyclines daunorubicin and idarubicin inhibited the OCTN1-mediated uptake of cytarabine in a concentration-dependent manner in various acute myeloid leukemia cell lines. In addition, it was determined that both daunorubicin and idarubicin inhibit cytarabine uptake in various acute myeloid leukemia cell lines [250].

#### 6.1.5. Tumor Microenvironment Impact on SLC Drug Transporter Expression and Function

Compared to normal conditions, the tumor microenvironment composed of endothelial cells, fibroblasts, perivascular cells and inflammatory cells is more prone to malignant cell proliferation, motility and adhesion. While the tumor microenvironment releases extracellular matrix proteins, growth factors and cytokines to support malignant cell growth, the tumors themselves produce growth factors and proteases which can modify their local microenvironment in order to make it more permissive for cell motility and adhesion. In the tumor microenvironment, the rapidly and continuously proliferating cells within solid tumors require a high oxygen supply, which can be limited by an undeveloped and dysfunctional vascular network throughout the tumor [251,252]. A decreased oxygen availability, defined as hypoxia, is generally associated with pathological conditions such as cancer, and can be chronic, resulting from limitations in the diffusion of oxygen to cells distal from the vessel, or acute, caused by a limited perfusion of oxygen delivery to adjacent cells [252]. Cellular adaptation to hypoxia is mainly mediated by a transcriptional regulator, such as hypoxia-inducible factors (HIFs), which induce a number of specific target genes [253]. HIFs act as heterodimers, consisting of an α subunit regulated by oxygen, and an oxygen-independent β subunit (called aryl hydrocarbon receptor nuclear translocator) [254]. In hypoxic conditions, the master transcriptional factor, hypoxia-inducible factor-1alpha (HIF-1α), is activated in order to regulate either the cell adaption or apoptosis via impacting the expression of various genes involved in metabolism, erythropoiesis, angiogenesis, cell proliferation and apoptosis [255,256,257]. To support glycolytic pathways, hypoxia induces alterations in the expression of several SLC transporters to ensure nutrient requirements to be maintained. For example, in hypoxic conditions, glucose transporters GLUT1 (SLC2A1) and GLUT3 (SLC2A3) are up-regulated to enhance glucose uptake in support of the hypoxia-induced glycolytic shift [258,259]. In addition, the HIF-1α-mediated up-regulation of monocarboxylate transporter 4 (MCT4, SLC16A3) has also been observed in hypoxia to enable the removal of intracellular lactate converted from pyruvate [260]. In contrast, several SLC transporters mediating drug uptake have been shown to be down-regulated in hypoxic conditions. For example, the expression of ENT1 and ENT2 was down-regulated in hypoxia through an HIF-1α-mediated mechanism [261,262]. In addition, the mRNA expression of OATP3A1 and OCT1 was decreased in BT474 estrogen receptor positive breast cancer cells exposed to chronic hypoxia compared to normoxia [263].

Moreover, the mRNA expression of SLCO1B3 and SLCO2B1 has been found to be induced by HIF-1α stabilizers and reduced by HIF-1α knockdown [264]. HIF-1α stabilization leads to cancer cell acclimatization to hypoxic conditions, increased proliferation, avoidance of apoptosis and therapy resistance in several cancer types [265]. Due to this, a strategy where the HIF-1α stabilization-mediated increase of OATP expression is used to enhance the drug accumulation to cancer cells likely would not lead to a more efficient therapy response. In contrast with several other cancer cells, it has been reported that, in pancreatic ductal adenocarcinoma cells HIF-1α, activation can act as a tumor suppressor [266]. However, OATP transporters have a high expression in pancreatic cancer, making HIF-1α induction an ineffective approach to increase anticancer drug accumulation [267].

In addition to hypoxia, inflammation is considered as a key characteristic of cancer and is closely associated with all stages of the development and progression of most cancer types [268,269,270]. Acute inflammation induces an anti-tumor immune response by promoting the maturation and function of dendritic cells and the initiation of effector T cells [271], and leads to cancer cell death. In contrast, the chronic inflammation involved in immunosuppression provides a favored microenvironment for tumorigenesis, development and metastasis [272]. Moreover, the inflammatory tumor microenvironment is a crucial factor for the therapeutic efficacy of conventional chemotherapy and immunotherapy [15].

In inflammation, both acute and chronic, cytokines play a major role in the modulation of gene expression. The cytokines released to bloodstream can interact with membrane receptors on epithelial or endothelial cells and induce a complex signaling cascade, resulting in a transduction of signal to the nucleus. The signal influences nuclear receptors, such as the PXR or CAR, acting as transcription factors regulating the expression of several genes including SLC transporters. Several studies showed the effect of cytokines on SLC expression. For instance, Vee et al. (2008) demonstrated that the mRNA and protein expression of the sodium-taurocholate co-transporting polypeptide (NTCP) and OATP1B1, as well as the mRNA expression of SLCO1B3, SLCO2B1, SLC22A11 and SLC22A7, were down-regulated after exposure to tumor necrosis factor alfa (TNF- α) and interleukin 6 (IL-6) in primary human hepatocytes. In addition, the activities of NTCP, OATP and OCT1 transporters were deceased after 48 h of exposure to TNF- α or IL-6 in primary human hepatocytes [273]. Similarly, treatment with IL-1β lead to the down-regulation of the mRNA and protein expression as well as the activity of NTCP in HepaRG cells. The SLCO2B1 (mRNA), OATP1B1 (mRNA and protein) and SLCO1B3 (mRNA) expression was also reduced in human primary hepatocytes treated with IL-1β for 24 h [274]. An anti-inflammatory therapeutic approach to the prevention and treatment of cancer has been widely investigated in preclinical and clinical settings [269]. However, the targeting of inflammatory pathways to modulate SLC transporter expression in cancer has not been studied and can be a promising approach to overcoming drug resistance due to a low SLC transporter expression.

The high rates of glycolysis and lactic acid secretion due to the altered metabolism in cancer cells affects the extracellular pH in the tumor microenvironment making it acidic compared to normal tissues [275]. The low pH of tumor microenvironment can affect the function of proton-coupled transporters by creating a higher membrane potential across the cancer cell membrane. It has been reported that the PCFT (SLC46A1)-mediated transport of methotrexate is higher at pH lower than 7 compared to pH 7.4 [22]. In addition, the low pH can broaden the substrate specificity of OATP2B1 [276]. For example, OATP1B3 substrate fexofenadine can be transported by OATP2B1 in acidic pH. However, it is not known whether any anticancer drugs would become OATP2B1 substrates at acidic pH.

### 6.2. Drug Delivery via Transporters Highly Expressed in Cancer Cells and Exploiting the Cancer Dependence of Transporters

The metabolic alterations in cancer cells due to oncogene signaling result in changes in the SLC drug transporter expression, and the changes in the SLC drug transporter expression may affect drug accumulation to the cancer cells, influencing the therapeutic response. The SLC drug expression can differ greatly between the cancerous cells and the adjacent healthy tissue, as can be seen in Table 1. The down-regulation of SLC drug transporters in cancer cells and the high expression in the cells responsible for the absorption, distribution, metabolism and elimination of drugs, sets a challenge for efficient drug delivery in the target cells. The oncogenic regulation of metabolic reprogramming and the Warburg effect induce the expression of the transporters required to supply energy and nutrients for the rapid growth of cancer cells [152,153]. Furthermore, the increased metabolic rate of the cancer cells demands a more efficient transporter-mediated removal of toxic metabolites and a reduction of oxidative stress [277]. The highly expressed transporters involved in the altered energy metabolism in cancer cells provide drug delivery opportunities (Table 2). The majority of the SLC transporters are not considered to be drug transporters, and thus, for their exploitation for anticancer drug delivery, prodrug and nanoparticle technologies have been developed (Table 2), which are discussed below.

#### 6.2.1. Glucose Transporter 1 (GLUT1 Encoded by SLC2A1)

GLUT1 (SLC2A1) has remained the main focus of cancer research, as this transporter plays a crucial role in the accelerated glucose uptake in cancer cells [278]. GLUT1 is ubiquitously distributed in normal tissues and overexpressed in many tumors, such as hepatic, brain, renal, pancreatic, lung, breast, esophageal, endometrial, ovarian, colorectal and cervical cancers [279]. The mechanisms of GLUT1 expression regulation in cancer are discussed in recent review [317]. GLUT1 expression is correlated with tumor development, and with adverse prognostic factors, such as poor differentiation and advanced tumor stage. For example, a high expression of GLUT1 was shown to be associated with poor survival in such cancer types as papillary thyroid carcinoma and stage I non-small cell lung carcinoma [318]. The GLUT1 expression levels have been considered as a marker of hypoxia, which characterizes more malignant tumors with a poor prognosis [319,320]. Due to all of these features, GLUT1 targeting has been successfully used for the tumor-specific delivery of imaging probes for in vivo tumor diagnosis by positron emission tomography and drug delivery to tumors overexpressing this transporter [321,322].

The high expression of GLUT1 at the blood–brain barrier endothelial cells and in glioma cells was used for GLUT1-mediated drug delivery using nanoparticles to treat glioma. In a study by Jiang et al. (2014), 2-deoxy-d-glucose modified poly(ethylene glycol)-co-poly(trimethylene carbonate) nanoparticles (dGlu-NP), developed to target GLUT1 transporter, demonstrated a higher uptake in rat glioma cells RG-2, which was inhibited by glucose. In addition, dGlu-NP showed higher accumulation in glioma compared to in the surrounding normal tissue and demonstrated a greater anti-glioblastoma efficacy of paclitaxel loaded to dGlu–NP compared to non-targeted nanoparticles or Taxol in orthotope glioma-bearing mice [280]. In another study, the authors developed D-glucosamine-conjugated paclitaxel-loaded poly(ethylene glycol)-co-poly(trimethylene carbonate) copolymer nanoparticles (DGlu–NP/PTX) to target glucose transporter. DGlu-NP/PTX demonstrated high anti-glioma efficacy in orthotope glioma-bearing mice compared to non-conjugated nanoparticles or Taxol [281]. Interestingly, Shao et al. (2014) targeted GLUT1 using the conjugation of polymeric micelles with dehydroascorbic acid (DHA) which is reduced to ascorbic acid inside the cells and, thus, enables the unidirectional transport of micelles to the cells because, when this GLUT1 substrate gets into cells, it is reduced into ascorbic acid and gets trapped within the cells [282]. The DHA-conjugated micelles loaded with paclitaxel demonstrated glucose transporter-mediated accumulation in the human-derived malignant glioma cells U87 cells and increased the survival time of glioma-bearing mice compared to non-targeted micelles or Taxol [282]. Patra et al. (2016) synthetized six isomers of glucose-platinum conjugates and revealed that C1α- and C2-substitution provide the highest GLUT1-mediated accumulation in DU145 prostate cancer cells. Importantly, the glucose-platinum conjugate substituted in the C2-position, which showed the greatest GLUT1 specific internalization and the highest cancer targeting ability, demonstrated anti-tumor efficacy and selective uptake in tumors with no observable toxicity in a syngeneic breast cancer mouse model overexpressing GLUT1 [283].

These examples demonstrate that utilization of glucose transporters such as GLUT1 can be a promising target for cancer drug delivery. However, the ubiquitous expression of the transporter in the normal tissues requires a careful evaluation of the biodistribution and the toxicity of the developed drug delivery systems.

#### 6.2.2. Monocarboxylate Transporters (MCTs)

MCTs are transporters of the SLC16A family consisting of 14 homologues in mammals [284]. The transporter mediates the proton-linked transport of monocarboxylates such as L-lactate, pyruvate and the ketone bodies across the plasma membrane. Among the MCTs, MCT1 (SLC16A1) and MCT4 (SLC16A3) have been most extensively studied in cancer, as these transporters play important role in lactate transport in cancer cells to support their survival [278,323]. MCT1 has a high affinity for lactate and is considered the major player in the lactate uptake of malignant cells which use lactate to fuel oxidative phosphorylation. In contrast, MCT4 possess a lower affinity for lactate than MCT1 and mainly exports lactate from hypoxic cancer cells [324,325]. MCT1 and MCT4 have been found to be dramatically up-regulated and associated with a poor prognosis in multiple malignant tumors, such as prostate cancer, lymphoma, peritoneal carcinomatosis and oral cavity cancer [285,286,287,326]. Therefore, the transporters represent attractive targets to deliver anticancer drugs.

Venishetty et al. (2013) conjugated β-hydroxybutyric acid, MCT1 substrate and solid lipid nanoparticles loaded with docetaxel to enhance brain delivery and demonstrated an MCT1-mediated increased delivery of docetaxel in brain endothelial cells as compared to Taxotere or non-targeted nanoparticles [288]. Interestingly, the involvement of MCT1 in the uptake of the anticancer drug 3-bromopyruvate in cancer cells has been identified by a genome-wide genetic screening [289]. The lethal concentration 50 (LC50) of 3-bromopyruvate correlated to the levels of MCT1 expression in acute promyelocytic leukemia (HL60), acute monocytic leukemia (THP1) and acute erythroid leukemia cell lines [327]. These studies indicate that MCT1 can be a promising candidate for the targeted delivery of anticancer drugs.

#### 6.2.3. Amino Acid Transporters

Rapid cancer cell growth is dependent on a homeostatic concentration of cytosolic amino acids [328], providing an attractive drug target and opportunity for drug delivery. However, due to substrate specificity, the design of anticancer drugs capable of utilizing amino acid transporters for cell uptake can be challenging [329]. Therefore, prodrugs and nanoparticles have been developed as an attempt to mediate drug delivery to cancer cells via amino acid transporters.

In addition to the drugs currently in clinical use, there are numerous published prodrugs and nanoparticles that aim at utilizing LAT1 for anticancer drug delivery. Recently, we covered information about the development of LAT1-utilizing compounds and nanoparticles targeting cancer cells in cancers [164,291]. In addition to the reports discussed in Puris et al. (2020 and 2022), several new attempts to target LAT1 for cancer delivery have been developed. Kaneda-Nakashima et al. (2021) conjugated radionucleotide Astatine-211 [^211^At] with α-methyl-L-tyrosine (AMT) as a LAT1-carrier to cancer cells [330]. The compound demonstrated LAT1-mediated accumulation in human pancreatic cancer (PANC-1) cells [330]. However, although the compound showed high accumulation in the tumor in the tumor-bearing mice at 1 h after I.V. injection, the distribution to the kidneys and pancreas was equally high [330]. Similar results were shown for a LAT1-selective α-radionuclide-labelled amino acid analogue, 2-[^211^At]astato-α-methyl-L-phenylalanine (2-[^211^At]AAMP) [292]. Wang et al. (2020) targeted LAT1 and ATB^0,+^ (SLC6A14) by using tyrosine conjugated to liposomes with PEG containing pH-sensitive aromatic imine bonds [293]. These dual-targeted liposomes showed higher accumulation after 12 and 14 h of incubation in human breast cancer MCF-7, BxPC-3 and NIH/3T3 cells compared to non-targeted liposomes [293]. The dual-targeted liposomes loaded with irinotecan showed the highest tumor accumulation and anti-tumor efficacy in tumor-bearing BxPC-3 Balb/c-nu mice compared to non-targeted liposomes and non-sensitive liposomes [293]. However, the drug was highly distributed to other organs such as the liver and spleen after the administration of the dual-targeted liposome in vivo. Moreover, the distribution and efficacy of the developed liposomes was not compared to irinotecan itself. In another study, L-tyrosine ester- and amide-conjugates of chlorambucil were synthetized and demonstrated binding to LAT1 in the human breast cancer MCF-7 cell line, while the distribution in vivo of these derivatives has not been evaluated [294].

Alanine, Serine, Cysteine Transporter 2 (ASCT2), encoded by SLC1A5, mediates a Na^+^-dependent exchange of glutamine, which is known to drive the growth and proliferation of tumor cells, in the antiport of serine, threonine or asparagine, with a questionable role for cysteine as a substrate [331]. Cancer cells display metabolic reprogramming and a high demand for increased consumption of amino acids, particularly for glutamine [332]. ASCT2 is up-regulated in a variety of cancer types, including colon, kidney, liver, lung, ovarian, pancreatic, stomach and cutaneous cancers [300]. Due to its function as glutamine transporter, ASCT2 has been proposed as a pharmacological target for specifically inhibiting the growth and development of cancer cells [278,333,334].

Glutamine was conjugated to β-cyclodextrin (GLN-CD), which was used to prepare doxorubicin inclusion complexes (DOX@GLN-CD) for the treatment of triple-negative breast cancer [301]. The developed DOX@GLN-CD complexes showed an ASCT2-mediated accumulation in TNBC cells, such as MDA-MB-231 and BT549, and induced a G2/M blockade and apoptosis, while the accumulation of the complex in nontumorigenic MCF10A cells with a low expression of ASCT2 was not considerable. In MDA-MB-231 tumor-bearing mice, DOX@GLN-CD accumulated exclusively in tumors and suppressed tumor growth with minimized toxic effects compared to the same dose of doxorubicin itself. This study demonstrated that targeting ASCT2 can provide potential tools for drug delivery to cancer cells overexpressing this transporter. In another study, Wang et al. (2018) developed polyglutamine (PGS), a glutamine macromolecular analogue, for siRNA delivery to cancer cells. The SLC1A5-mediated delivery of the PGS/siRNA complex was shown in cisplatin-resistant human lung adenocarcinoma A549/DDP cells significantly overexpressing SLC1A5 [302]. In a lung orthotopic tumor mouse model, the complex PGS/siRNA predominantly accumulated in the lungs, which possess high expression of SLC1A5, and decreased the tumor growth.

Amino acid transporter B^0,+^ (ATB^0,+^), encoded by SLC6A14, transports one molecule of neutral (index “0”) or basic (index “+”) amino acids in a symport with 2 Na^+^ and 1 Cl [303]. The expression of SLC6A14 is significantly increased in several types of human cancers, in particular in solid tumors, with the highest up-regulation in colorectal, pancreatic and cervical cancer [304,305,335]. Several attempts to develop amino acid conjugates for enhanced drug delivery via ATB^0,+^ to tumors have been made. For example, liposomes functionalized with lysine and polyoxyethylene stearate conjugate (LPS) to target ATB^0,+^ were developed [306]. These liposomes loaded with docetaxel delivered the drug to the tumor site in mice bearing a murine hepatoma tumor (H_22_) with consequent greater anti-tumor efficacy and less systemic toxicity. Later, Luo et al. (2017) developed liposomes functionalized with aspartate-polyoxyethylene stearate conjugate (APS) for targeted ATB^0,+^-mediated delivery of docetaxel to ATB^0,+^-overexpressing human lung cells, A549 cells [316]. Compared with conventional liposomes, APS-liposomes demonstrated an increased ATB^0,+^-mediated intracellular accumulation of docetaxel in human lung cancer A549 cells [307]. Kou et al. (2020) developed lysine-conjugated liposomes and demonstrated their binding to ATB^0,+^ in MCF7 cells [308]. The lysine-conjugated liposomes increased the uptake and cytotoxicity of gemcitabine in MCF7 cells in an ATB^0,+^-dependent manner. These reports demonstrate that ATB^0,+^ could be exploited for targeted drug delivery for enhanced cancer therapy.

#### 6.2.4. Proton-Coupled Peptide Transporter 1

Proton-coupled peptide transporter 1 (PEPT1), encoded by SLC15A1, is a di- and tripeptide uptake transporter transporting its substrates with low affinity and high capacity [309]. PEPT1 is mainly expressed on the apical microvilli of enterocytes in the small intestine. However, it has been shown that the transporter is highly expressed in patient-derived prostate cancer cells and in hepatocellular carcinoma [310,311]. Interestingly, Schniers et al. (2021) showed in patient-derived cells and a xenograft mouse model of pancreatic adenocarcinoma that PEPT1 is overexpressed and essential for tumor growth, making it a potential drug target [311]. In addition, the high PEPT1 expression and the cancer’s dependency on the transporter makes it an interesting target for anticancer drug delivery. None of the currently available anticancer drugs are substrates of PEPT1, but several prodrugs and anticancer-drug-loaded nanoparticles targeting PEPT1 for enhanced oral bioavailability or cancer cell uptake have been published [312,313,314,315,316].

In a study by Landowsky et al. (2005), a series of amino acid ester prodrugs of floxuridine were synthesized and investigated for their ability to utilize PEPT1 for cell uptake and enhance the parent drug efficacy [315]. The results showed an increased cell uptake and antiproliferative efficacy for prolyl and lysyl floxuridine prodrugs in MDCK/PEPT1 cells compared with MDCK control cells. A PEPT1 targeted prodrug has been developed to increase the cell accumulation of doxorubicin and its efficacy in liver cancer cells [316]. Doxorubicin was conjugated with the PEPT1 substrate Gly-Gly-Gly, followed by the investigation of the anti-tumor effect of Doxorubicin-tripeptide conjugate in liver cancer cells and xenografts. The anti-tumor effects of the doxorubicin-tripeptide conjugate showed a more effective anticancer treatment in vitro and in vivo with lower toxicity in vivo compared to doxorubicin itself. However, the role of PEPT1 in the elevated cell uptake and efficacy was not determined unequivocally.

## 7. Conclusions and Future Perspectives

SLC transporters play a major role in the cell accumulation of anticancer drugs and thus in the efficacy of the drug treatment. A low expression of drug influx transporters in cancer cells leads to therapy resistance, as the drug concentrations in the target cells are decreased, and increasing the dose is not possible due to drug accumulation to healthy tissues leading to unbearable adverse effects. On the other hand, knowledge of the influx SLC transporters expressed in cancer cells presents a means of delivering anticancer drugs efficiently to their targets. However, as indicated in our review, there is a great need to collect more quantitative expression data on transporter expression in different cancers, as there are currently only handful of reports available, namely on hepatocellular carcinoma, colorectal carcinoma and the liver metastasis of colorectal cancer. Moreover, more research has to be undertaken to elucidate the possible differences in transporter expression between cancer subtypes, as cancers driven by oncogene or tumor suppressor gene mutations may have significant differences in their drug transporter expression. This would allow us to better understand how transporter expression is controlled in different cancer subtypes and would present potential clues about how to selectively regulate drug transporter expression for enhanced anticancer drug delivery. Knowledge of SLC transporters mediating drug influx and quantitative transporter protein expression data in cancer cells would provide an opportunity for the estimation of drug delivery efficacy to the target cells by the means of physiologically based pharmacokinetic modeling. This in turn would help tremendously in the selection and development of efficient anticancer drug therapies. Moreover, quantitative information on transporter expression in cancers could be used in the rational design of novel drug delivery strategies such as the modulation of drug transporter expression or the utilization of highly expressed nutrient transporters using transporter-targeted prodrugs and nanoparticles. Importantly, for many anticancer drugs, the SLC transporters responsible for the cell uptake are unknown. Therefore, extensive work has to be performed in the field of transporter research to gain more knowledge of the SLC transporters delivering anticancer drugs to cancer cells, in order to fully take advantage of the information about transporter expression. In addition to investigating transporter expression, an analysis of the dependency of different cancers on specific transporters is also of great interest, as it would allow the targeting of drugs to transporters that cancer cannot down-regulate, thus avoiding low drug influx-mediated drug resistance. For example, there are reports available revealing the dependency of certain cancers on glucose or amino acid transporters. However, research is needed in order to reveal more potential targetable transporters in different cancers.

As presented in this review, several strategies for increasing the cancer cell delivery of anticancer drugs have been investigated. However, none of the reviewed drug delivery strategies are currently in clinical use and there is a lot of work yet to be performed to reveal the potential of these strategies. Firstly, a more accurate analysis is needed to determine whether poor drug efficacy is indeed caused by low transporter expression and the resulting low drug influx before the drug delivery strategies are applied. In the majority of the published prodrug and nanoparticle delivery strategies, it was not clear whether the poor influx of the selected anticancer drug against the investigated cancer was the efficacy-limiting mechanism. In addition, the majority of the studies were performed in cancer cell lines, whose relevance in terms of transporter expression compared to clinical cancers is questionable or unknown. Secondly, for the majority of the published prodrugs and nanoparticles, no thorough pharmacokinetic analysis and in vivo tumor accumulation study of the anticancer drug was reported. The lack of these experiments hinders the evaluation of whether the anticancer drug delivery to the target site was significantly improved compared to the anticancer drug dosing.

From the different strategies for influencing drug influx transporter expression in cancer cells, the use of FXR agonists, CAR antagonists and PKCs inhibitors show promise and should be investigated more thoroughly. According to current research, by affecting these targets, it is possible to increase the drug influx transporter expression in cancer cells without simultaneously increasing the efflux transporter expression. In addition, PKCs inhibitors have been shown, in vitro, to affect transporter expression selectively in cancer cells over hepatocytes. However, more studies should be made in order to determine the potential beneficial effects on anticancer drug accumulation into tumors and the consequent drug efficacy. Importantly, the effect of FXR, CAR and PKCs ligands on anticancer drug pharmacokinetics and accumulation to healthy tissues should be investigated thoroughly.

In summary, despite the recent advances in the investigation of SLC transporters, there are still significant gaps in our understanding of their role in anticancer drug delivery and poor drug efficacy caused by low drug influx transporter expression. However, the innovative strategies proposed for enhanced transporter-mediated anticancer drug delivery, when thoroughly investigated, show promise for overcoming transporter-mediated anticancer drug resistance and facilitating the development of effective anticancer drug treatments.

## Figures and Tables

**Table 2:** Highly expressed SLC transporters, which have been utilized to improve anticancer drug delivery to cancer cells.

Gene Name	Protein Name	Natural Substrates	Tissue Expression	High Expression in Cancer	Utilization in Anticancer Drug Delivery	References
SLC2A1	GLUT1	Glucose, galactose, mannose, glucosamine	Erythrocytes, brain, (endothelial cells), blood-tissue barrier, several fetal tissues	Liver, brain, renal, pancreatic, lung, breast, esophageal, endometrial, ovarian, colorectal and cervical cancers	dGlu-conjugated nanoparticles, D-glucosamine-conjugated nanoparticles, DHA-conjugated micelles, polymeric micelles. glucose-platinum conjugates	[35,278,279,280,281,282,283]
SLC16A1	MCT1	lactate, pyruvate, ketone bodies	Ubiquitous	Prostate cancer, lymphoma, peritoneal carcinomatosis and oral cavity cancer	β-hydroxybutyric acid -conjugated nanoparticles, 3-bromopyruvate	[35,284,285,286,287,288,289,290]
SLC7A5	LAT1	Phenylalanine, leucine, tryptophan	Brain (endothelial cells), testis, retina, esophagus, testis, placenta and bone marrow	Colorectal cancer, gliomablastoma, triple-negative breast cancer and HER2-positive breast cancers and MYC driver ER-positive breast cancer	L-phenylalanine prodrug of melphalan, threonine-derivative of gemcitabine, aspartate derivative of doxorubicin, liposomes of L-Dopa functionalized amphiphile, lysine-conjugated methotrexate, L- and D-Dopa conjugated anisotropic gold nanoparticles, lysine- and aromatic amino acid-mustards, α-methyl-L-tyrosine conjugate of Astatine-211, tyrosine-conjugated liposomes, L-tyrosine ester- and amide-conjugates of chlorambucil	[35,164,290,291,292,293,294,295,296,297,298,299]
SLC1A5	ASCT2	L-alanine, L-serine, L-threonine, L-glutamine, L- asparagine	Lung, skeletal muscle, large intestine, kidney, testis, adipose tissue	Colon, kidney, liver, lung, ovarian, pancreatic, stomach and cutaneous cancers	Glutamine-conjugated β-cyclodextrin inclusion complexes of doxorubicin, polyglutamine for siRNA delivery	[35,300,301,302]
SLC6A14	ATB^0,+^	Neutral, cationic amino acids	Lung, trachea, salivary gland, mammary gland, stomach, pituitary gland, intestine, uterus, prostate, testis	Colorectal, pancreatic and cervical cancer	Lysine-conjugated liposomes, lysine and polyoxyethylene stearate -conjugated liposomes, aspartate-polyoxyethylene stearate -conjugated liposomes,	[35,303,304,305,306,307,308]
SLC15A1	PEPT1	Di- and tripeptides, protons, beta-lactam antibiotics	Small intestine, kidney, pancreas, bile duct, liver	Prostate cancer, hepatocellular carcinoma, pancreatic adenocarcinoma	Prolyl and lysyl floxuridine prodrugs, Gly-Gly-Gly conjugate of doxorubicin	[35,309,310,311,312,313,314,315,316]

dGlu–2-deoxy-d-glucose; DHA–dehydroascorbic acid.

## Data Availability

Not applicable.

## References

[1] Ferlay J., Colombet M., Soerjomataram I., Parkin D.M., Pineros M., Znaor A., Bray F. (2021). Cancer statistics for the year 2020: An overview. Int. J. Cancer.

[2] Bray F., Moller B. (2006). Predicting the future burden of cancer. Nat. Rev. Cancer.

[3] Falzone L., Salomone S., Libra M. (2018). Evolution of Cancer Pharmacological Treatments at the Turn of the Third Millennium. Front. Pharmacol..

[4] Tsimberidou A.M. (2015). Targeted therapy in cancer. Cancer Chemother. Pharmacol..

[5] Gottesman M.M., Pastan I. (1993). Biochemistry of multidrug resistance mediated by the multidrug transporter. Annu. Rev. Biochem..

[6] Longley D.B., Johnston P.G. (2005). Molecular mechanisms of drug resistance. J. Pathol..

[7] Cree I.A., Charlton P. (2017). Molecular chess? Hallmarks of anti-cancer drug resistance. BMC Cancer.

[8] Fletcher J.I., Haber M., Henderson M.J., Norris M.D. (2010). ABC transporters in cancer: More than just drug efflux pumps. Nat. Rev. Cancer.

[9] Fletcher J.I., Williams R.T., Henderson M.J., Norris M.D., Haber M. (2016). ABC transporters as mediators of drug resistance and contributors to cancer cell biology. Drug Resist. Updat..

[10] Li W., Zhang H., Assaraf Y.G., Zhao K., Xu X., Xie J., Yang D.H., Chen Z.S. (2016). Overcoming ABC transporter-mediated multidrug resistance: Molecular mechanisms and novel therapeutic drug strategies. Drug Resist. Updat..

[11] Sutherland R., Meeson A., Lowes S. (2020). Solute transporters and malignancy: Establishing the role of uptake transporters in breast cancer and breast cancer metastasis. Cancer Metastasis Rev..

[12] Wu G., Wilson G., George J., Liddle C., Hebbard L., Qiao L. (2017). Overcoming treatment resistance in cancer: Current understanding and tactics. Cancer Lett..

[13] Juliano R.L., Ling V. (1976). A surface glycoprotein modulating drug permeability in Chinese hamster ovary cell mutants. Biochim. Biophys. Acta.

[14] Holohan C., Van Schaeybroeck S., Longley D.B., Johnston P.G. (2013). Cancer drug resistance: An evolving paradigm. Nat. Rev. Cancer.

[15] Vasan N., Baselga J., Hyman D.M. (2019). A view on drug resistance in cancer. Nature.

[16] Gottesman M.M. (2002). Mechanisms of cancer drug resistance. Annu. Rev. Med..

[17] Aleksakhina S.N., Kashyap A., Imyanitov E.N. (2019). Mechanisms of acquired tumor drug resistance. Biochim. Biophys. Acta Rev. Cancer.

[18] Fojo T., Bates S. (2003). Strategies for reversing drug resistance. Oncogene.

[19] Hayashi T., Konishi I. (2021). Correlation of anti-tumour drug resistance with epigenetic regulation. Br. J. Cancer.

[20] Taylor S.T., Hickman J.A., Dive C. (2000). Epigenetic determinants of resistance to etoposide regulation of Bcl-X(L) and Bax by tumor microenvironmental factors. J. Natl. Cancer Inst..

[21] Dagogo-Jack I., Shaw A.T. (2018). Tumour heterogeneity and resistance to cancer therapies. Nat. Rev. Clin. Oncol..

[22] Anderson C.M., Thwaites D.T. (2010). Hijacking solute carriers for proton-coupled drug transport. Physiology.

[23] Dean M., Hamon Y., Chimini G. (2001). The human ATP-binding cassette (ABC) transporter superfamily. J. Lipid Res..

[24] Borst P., Elferink R.O. (2002). Mammalian ABC transporters in health and disease. Annu. Rev. Biochem..

[25] Dunbar L.A., Caplan M.J. (2000). The cell biology of ion pumps: Sorting and regulation. Eur. J. Cell Biol..

[26] Robey R.W., Pluchino K.M., Hall M.D., Fojo A.T., Bates S.E., Gottesman M.M. (2018). Revisiting the role of ABC transporters in multidrug-resistant cancer. Nat. Rev Cancer.

[27] Xiao H., Zheng Y., Ma L., Tian L., Sun Q. (2021). Clinically-Relevant ABC Transporter for Anti-Cancer Drug Resistance. Front. Pharmacol..

[28] Navale A.M., Paranjape A.N. (2016). Glucose transporters: Physiological and pathological roles. Biophys. Rev..

[29] Mueckler M., Thorens B. (2013). The SLC2 (GLUT) family of membrane transporters. Mol. Aspects Med..

[30] Kim Y.H., Jeong D.C., Pak K., Han M.E., Kim J.Y., Liangwen L., Kim H.J., Kim T.W., Kim T.H., Hyun D.W. (2017). SLC2A2 (GLUT2) as a novel prognostic factor for hepatocellular carcinoma. Oncotarget.

[31] Daskalow K., Pfander D., Weichert W., Rohwer N., Thelen A., Neuhaus P., Jonas S., Wiedenmann B., Benckert C., Cramer T. (2009). Distinct temporospatial expression patterns of glycolysis-related proteins in human hepatocellular carcinoma. Histochem. Cell Biol..

[32] Godoy A., Ulloa V., Rodriguez F., Reinicke K., Yanez A.J., Garcia Mde L., Medina R.A., Carrasco M., Barberis S., Castro T. (2006). Differential subcellular distribution of glucose transporters GLUT1-6 and GLUT9 in human cancer: Ultrastructural localization of GLUT1 and GLUT5 in breast tumor tissues. J. Cell Physiol..

[33] Christensen H.N. (1990). Role of amino acid transport and countertransport in nutrition and metabolism. Physiol. Rev..

[34] Oxender D.L., Christensen H.N. (1963). Evidence for two types of mediation of neutral and amino-acid transport in Ehrlich cells. Nature.

[35] Fagerberg L., Hallstrom B.M., Oksvold P., Kampf C., Djureinovic D., Odeberg J., Habuka M., Tahmasebpoor S., Danielsson A., Edlund K. (2014). Analysis of the human tissue-specific expression by genome-wide integration of transcriptomics and antibody-based proteomics. Mol. Cell Proteom..

[36] Wisniewski J.R., Dus-Szachniewicz K., Ostasiewicz P., Ziolkowski P., Rakus D., Mann M. (2015). Absolute Proteome Analysis of Colorectal Mucosa, Adenoma, and Cancer Reveals Drastic Changes in Fatty Acid Metabolism and Plasma Membrane Transporters. J. Proteome Res..

[37] Wisniewski J.R., Ostasiewicz P., Dus K., Zielinska D.F., Gnad F., Mann M. (2012). Extensive quantitative remodeling of the proteome between normal colon tissue and adenocarcinoma. Mol. Syst. Biol..

[38] Nawashiro H., Otani N., Uozumi Y., Ooigawa H., Toyooka T., Suzuki T., Katoh H., Tsuzuki N., Ohnuki A., Shima K. (2005). High expression of L-type amino acid transporter 1 in infiltrating glioma cells. Brain Tumor Pathol..

[39] El Ansari R., Craze M.L., Miligy I., Diez-Rodriguez M., Nolan C.C., Ellis I.O., Rakha E.A., Green A.R. (2018). The amino acid transporter SLC7A5 confers a poor prognosis in the highly proliferative breast cancer subtypes and is a key therapeutic target in luminal B tumours. Breast Cancer Res..

[40] Maimaiti M., Sakamoto S., Yamada Y., Sugiura M., Rii J., Takeuchi N., Imamura Y., Furihata T., Ando K., Higuchi K. (2020). Expression of L-type amino acid transporter 1 as a molecular target for prognostic and therapeutic indicators in bladder carcinoma. Sci. Rep..

[41] Yanagisawa N., Ichinoe M., Mikami T., Nakada N., Hana K., Koizumi W., Endou H., Okayasu I. (2012). High expression of L-type amino acid transporter 1 (LAT1) predicts poor prognosis in pancreatic ductal adenocarcinomas. J. Clin. Pathol..

[42] Lin J., Raoof D.A., Thomas D.G., Greenson J.K., Giordano T.J., Robinson G.S., Bourner M.J., Bauer C.T., Orringer M.B., Beer D.G. (2004). L-type amino acid transporter-1 overexpression and melphalan sensitivity in Barrett’s adenocarcinoma. Neoplasia.

[43] Greig N.H., Sweeney D.J., Rapoport S.I. (1987). Melphalan concentration dependent plasma protein binding in healthy humans and rats. Eur. J. Clin. Pharmacol..

[44] Goldman I.D. (1971). The characteristics of the membrane transport of amethopterin and the naturally occurring folates. Ann. N. Y. Acad. Sci..

[45] Goldman I.D., Lichtenstein N.S., Oliverio V.T. (1968). Carrier-mediated transport of the folic acid analogue, methotrexate, in the L1210 leukemia cell. J. Biol. Chem..

[46] Sierra E.E., Brigle K.E., Spinella M.J., Goldman I.D. (1997). pH dependence of methotrexate transport by the reduced folate carrier and the folate receptor in L1210 leukemia cells. Further evidence for a third route mediated at low pH. Biochem. Pharmacol..

[47] Alam C., Hoque M.T., Finnell R.H., Goldman I.D., Bendayan R. (2017). Regulation of Reduced Folate Carrier (RFC) by Vitamin D Receptor at the Blood-Brain Barrier. Mol. Pharm..

[48] Westerhof G.R., Schornagel J.H., Kathmann I., Jackman A.L., Rosowsky A., Forsch R.A., Hynes J.B., Boyle F.T., Peters G.J., Pinedo H.M. (1995). Carrier- and receptor-mediated transport of folate antagonists targeting folate-dependent enzymes: Correlates of molecular-structure and biological activity. Mol. Pharmacol..

[49] Zhang X., Zhang D., Huang L., Li G., Chen L., Ma J., Li M., Wei M., Zhou W., Zhou C. (2019). Discovery of Novel Biomarkers of Therapeutic Responses in Han Chinese Pemetrexed-Based Treated Advanced NSCLC Patients. Front. Pharmacol..

[50] Lau D.T., Flemming C.L., Gherardi S., Perini G., Oberthuer A., Fischer M., Juraeva D., Brors B., Xue C., Norris M.D. (2015). MYCN amplification confers enhanced folate dependence and methotrexate sensitivity in neuroblastoma. Oncotarget.

[51] Odin E., Sonden A., Gustavsson B., Carlsson G., Wettergren Y. (2015). Expression of Folate Pathway Genes in Stage III Colorectal Cancer Correlates with Recurrence Status Following Adjuvant Bolus 5-FU-Based Chemotherapy. Mol. Med..

[52] Nunez M.I., Behrens C., Woods D.M., Lin H., Suraokar M., Kadara H., Hofstetter W., Kalhor N., Lee J.J., Franklin W. (2012). High expression of folate receptor alpha in lung cancer correlates with adenocarcinoma histology and EGFR [corrected] mutation. J. Thorac. Oncol..

[53] Siu M.K., Kong D.S., Chan H.Y., Wong E.S., Ip P.P., Jiang L., Ngan H.Y., Le X.F., Cheung A.N. (2012). Paradoxical impact of two folate receptors, FRalpha and RFC, in ovarian cancer: Effect on cell proliferation, invasion and clinical outcome. PLoS ONE.

[54] Hagenbuch B., Meier P.J. (2004). Organic anion transporting polypeptides of the OATP/SLC21 family: Phylogenetic classification as OATP/ SLCO superfamily, new nomenclature and molecular/functional properties. Pflugers. Arch..

[55] Vasilogianni A.M., Al-Majdoub Z.M., Achour B., Annie Peters S., Barber J., Rostami-Hodjegan A. (2022). Quantitative Proteomics of Hepatic Drug-Metabolizing Enzymes and Transporters in Patients With Colorectal Cancer Metastasis. Clin. Pharmacol. Ther..

[56] Meyer zu Schwabedissen H.E., Tirona R.G., Yip C.S., Ho R.H., Kim R.B. (2008). Interplay between the nuclear receptor pregnane X receptor and the uptake transporter organic anion transporter polypeptide 1A2 selectively enhances estrogen effects in breast cancer. Cancer Res..

[57] Tang T., Wang G., Liu S., Zhang Z., Liu C., Li F., Liu X., Meng L., Yang H., Li C. (2021). Highly expressed SLCO1B3 inhibits the occurrence and development of breast cancer and can be used as a clinical indicator of prognosis. Sci. Rep..

[58] Ballestero M.R., Monte M.J., Briz O., Jimenez F., Gonzalez-San Martin F., Marin J.J. (2006). Expression of transporters potentially involved in the targeting of cytostatic bile acid derivatives to colon cancer and polyps. Biochem. Pharmacol..

[59] Cooper E., Woolf Z., Swanson M.E.V., Correia J., Schweder P., Mee E., Heppner P., Turner C., Faull R.L.M., Scotter E.L. (2022). Single-cell image analysis reveals over-expression of organic anion transporting polypeptides (OATPs) in human glioblastoma tissue. Neurooncol. Adv..

[60] Billington S., Ray A.S., Salphati L., Xiao G., Chu X., Humphreys W.G., Liao M., Lee C.A., Mathias A., Hop C. (2018). Transporter Expression in Noncancerous and Cancerous Liver Tissue from Donors with Hepatocellular Carcinoma and Chronic Hepatitis C Infection Quantified by LC-MS/MS Proteomics. Drug Metab. Dispos..

[61] Svoboda M., Wlcek K., Taferner B., Hering S., Stieger B., Tong D., Zeillinger R., Thalhammer T., Jager W. (2011). Expression of organic anion-transporting polypeptides 1B1 and 1B3 in ovarian cancer cells: Relevance for paclitaxel transport. Biomed. Pharmacother..

[62] Kounnis V., Ioachim E., Svoboda M., Tzakos A., Sainis I., Thalhammer T., Steiner G., Briasoulis E. (2011). Expression of organic anion-transporting polypeptides 1B3, 1B1, and 1A2 in human pancreatic cancer reveals a new class of potential therapeutic targets. Onco Targets Ther..

[63] Wright J.L., Kwon E.M., Ostrander E.A., Montgomery R.B., Lin D.W., Vessella R., Stanford J.L., Mostaghel E.A. (2011). Expression of SLCO transport genes in castration-resistant prostate cancer and impact of genetic variation in SLCO1B3 and SLCO2B1 on prostate cancer outcomes. Cancer Epidemiol. Biomarkers Prev..

[64] Terakawa T., Katsuta E., Yan L., Turaga N., McDonald K.A., Fujisawa M., Guru K.A., Takabe K. (2018). High expression of SLCO2B1 is associated with prostate cancer recurrence after radical prostatectomy. Oncotarget.

[65] Koepsell H. (2013). The SLC22 family with transporters of organic cations, anions and zwitterions. Mol. Aspects Med..

[66] Neul C., Schaeffeler E., Sparreboom A., Laufer S., Schwab M., Nies A.T. (2016). Impact of Membrane Drug Transporters on Resistance to Small-Molecule Tyrosine Kinase Inhibitors. Trends Pharmacol. Sci..

[67] Koepsell H. (2004). Polyspecific organic cation transporters: Their functions and interactions with drugs. Trends Pharmacol. Sci..

[68] Jonker J.W., Schinkel A.H. (2004). Pharmacological and physiological functions of the polyspecific organic cation transporters: OCT1, 2, and 3 (SLC22A1-3). J. Pharmacol. Exp. Ther..

[69] Lautem A., Heise M., Grasel A., Hoppe-Lotichius M., Weiler N., Foltys D., Knapstein J., Schattenberg J.M., Schad A., Zimmermann A. (2013). Downregulation of organic cation transporter 1 (SLC22A1) is associated with tumor progression and reduced patient survival in human cholangiocellular carcinoma. Int. J. Oncol..

[70] Schaeffeler E., Hellerbrand C., Nies A.T., Winter S., Kruck S., Hofmann U., van der Kuip H., Zanger U.M., Koepsell H., Schwab M. (2011). DNA methylation is associated with downregulation of the organic cation transporter OCT1 (SLC22A1) in human hepatocellular carcinoma. Genome Med..

[71] Huang K.M., Leblanc A.F., Uddin M.E., Kim J.Y., Chen M., Eisenmann E.D., Gibson A.A., Li Y., Hong K.W., DiGiacomo D. (2020). Neuronal uptake transporters contribute to oxaliplatin neurotoxicity in mice. J. Clin. Investig..

[72] Sprowl J.A., Ciarimboli G., Lancaster C.S., Giovinazzo H., Gibson A.A., Du G., Janke L.J., Cavaletti G., Shields A.F., Sparreboom A. (2013). Oxaliplatin-induced neurotoxicity is dependent on the organic cation transporter OCT2. Proc. Natl. Acad. Sci. USA.

[73] Filipski K.K., Mathijssen R.H., Mikkelsen T.S., Schinkel A.H., Sparreboom A. (2009). Contribution of organic cation transporter 2 (OCT2) to cisplatin-induced nephrotoxicity. Clin. Pharmacol. Ther..

[74] Koepsell H., Schmitt B.M., Gorboulev V. (2003). Organic cation transporters. Rev. Physiol. Biochem. Pharmacol..

[75] Winter S., Fisel P., Buttner F., Rausch S., D’Amico D., Hennenlotter J., Kruck S., Nies A.T., Stenzl A., Junker K. (2016). Methylomes of renal cell lines and tumors or metastases differ significantly with impact on pharmacogenes. Sci. Rep..

[76] Gu J., Wang L., Li T., Tang S., Wang Y., Zhang W., Jiang X. (2019). Role and mechanism of organic cation transporter 3 in oxaliplatin treatment of colon cancer in vitro and in vivo. Oncol. Rep..

[77] Muller J., Lips K.S., Metzner L., Neubert R.H., Koepsell H., Brandsch M. (2005). Drug specificity and intestinal membrane localization of human organic cation transporters (OCT). Biochem. Pharmacol..

[78] Inazu M., Takeda H., Matsumiya T. (2003). Expression and functional characterization of the extraneuronal monoamine transporter in normal human astrocytes. J. Neurochem..

[79] Hayer-Zillgen M., Bruss M., Bonisch H. (2002). Expression and pharmacological profile of the human organic cation transporters hOCT1, hOCT2 and hOCT3. Br. J. Pharmacol..

[80] Sata R., Ohtani H., Tsujimoto M., Murakami H., Koyabu N., Nakamura T., Uchiumi T., Kuwano M., Nagata H., Tsukimori K. (2005). Functional analysis of organic cation transporter 3 expressed in human placenta. J. Pharmacol. Exp. Ther..

[81] Wu X., Kekuda R., Huang W., Fei Y.J., Leibach F.H., Chen J., Conway S.J., Ganapathy V. (1998). Identity of the organic cation transporter OCT3 as the extraneuronal monoamine transporter (uptake2) and evidence for the expression of the transporter in the brain. J. Biol. Chem..

[82] Hsu C.M., Lin P.M., Chang J.G., Lin H.C., Li S.H., Lin S.F., Yang M.Y. (2017). Upregulated SLC22A3 has a potential for improving survival of patients with head and neck squamous cell carcinoma receiving cisplatin treatment. Oncotarget.

[83] Yokoo S., Masuda S., Yonezawa A., Terada T., Katsura T., Inui K. (2008). Significance of organic cation transporter 3 (SLC22A3) expression for the cytotoxic effect of oxaliplatin in colorectal cancer. Drug Metab. Dispos..

[84] Namisaki T., Schaeffeler E., Fukui H., Yoshiji H., Nakajima Y., Fritz P., Schwab M., Nies A.T. (2014). Differential expression of drug uptake and efflux transporters in Japanese patients with hepatocellular carcinoma. Drug Metab. Dispos..

[85] Grundemann D., Harlfinger S., Golz S., Geerts A., Lazar A., Berkels R., Jung N., Rubbert A., Schomig E. (2005). Discovery of the ergothioneine transporter. Proc. Natl. Acad. Sci. USA.

[86] Pochini L., Scalise M., Galluccio M., Pani G., Siminovitch K.A., Indiveri C. (2012). The human OCTN1 (SLC22A4) reconstituted in liposomes catalyzes acetylcholine transport which is defective in the mutant L503F associated to the Crohn’s disease. Biochim. Biophys. Acta.

[87] Drenberg C.D., Gibson A.A., Pounds S.B., Shi L., Rhinehart D.P., Li L., Hu S., Du G., Nies A.T., Schwab M. (2017). OCTN1 Is a High-Affinity Carrier of Nucleoside Analogues. Cancer Res..

[88] Garrett Q., Xu S., Simmons P.A., Vehige J., Flanagan J.L., Willcox M.D. (2008). Expression and localization of carnitine/organic cation transporter OCTN1 and OCTN2 in ocular epithelium. Investig. Ophthalmol. Vis. Sci..

[89] Wada E., Koyanagi S., Kusunose N., Akamine T., Masui H., Hashimoto H., Matsunaga N., Ohdo S. (2015). Modulation of peroxisome proliferator-activated receptor-alpha activity by bile acids causes circadian changes in the intestinal expression of Octn1/Slc22a4 in mice. Mol. Pharmacol..

[90] Meetam P., Srimaroeng C., Soodvilai S., Chatsudthipong V. (2009). Regulatory role of testosterone in organic cation transport: In vivo and in vitro studies. Biol. Pharm. Bull..

[91] Pochini L., Galluccio M., Scalise M., Console L., Indiveri C. (2019). OCTN: A Small Transporter Subfamily with Great Relevance to Human Pathophysiology, Drug Discovery, and Diagnostics. SLAS Discov..

[92] Hu C., Lancaster C.S., Zuo Z., Hu S., Chen Z., Rubnitz J.E., Baker S.D., Sparreboom A. (2012). Inhibition of OCTN2-mediated transport of carnitine by etoposide. Mol Cancer Ther..

[93] Koepsell H., Endou H. (2004). The SLC22 drug transporter family. Pflugers. Arch..

[94] Wu X., Huang W., Prasad P.D., Seth P., Rajan D.P., Leibach F.H., Chen J., Conway S.J., Ganapathy V. (1999). Functional characteristics and tissue distribution pattern of organic cation transporter 2 (OCTN2), an organic cation/carnitine transporter. J. Pharmacol. Exp. Ther..

[95] Wang C., Uray I.P., Mazumdar A., Mayer J.A., Brown P.H. (2012). SLC22A5/OCTN2 expression in breast cancer is induced by estrogen via a novel intronic estrogen-response element (ERE). Breast Cancer Res. Treat..

[96] Fink M.A., Paland H., Herzog S., Grube M., Vogelgesang S., Weitmann K., Bialke A., Hoffmann W., Rauch B.H., Schroeder H.W.S. (2019). L-Carnitine-Mediated Tumor Cell Protection and Poor Patient Survival Associated with OCTN2 Overexpression in Glioblastoma Multiforme. Clin. Cancer Res..

[97] Anzai N., Kanai Y., Endou H. (2006). Organic anion transporter family: Current knowledge. J. Pharmacol. Sci..

[98] Jia Y., Liu Z., Wang C., Meng Q., Huo X., Liu Q., Sun H., Sun P., Yang X., Ma X. (2016). P-gp, MRP2 and OAT1/OAT3 mediate the drug-drug interaction between resveratrol and methotrexate. Toxicol. Appl. Pharmacol..

[99] Iwaki M., Shimada H., Irino Y., Take M., Egashira S. (2017). Inhibition of Methotrexate Uptake via Organic Anion Transporters OAT1 and OAT3 by Glucuronides of Nonsteroidal Anti-inflammatory Drugs. Biol. Pharm. Bull..

[100] Hosoyamada M., Sekine T., Kanai Y., Endou H. (1999). Molecular cloning and functional expression of a multispecific organic anion transporter from human kidney. Am. J. Physiol..

[101] Motohashi H., Sakurai Y., Saito H., Masuda S., Urakami Y., Goto M., Fukatsu A., Ogawa O., Inui K.I. (2002). Gene expression levels and immunolocalization of organic ion transporters in the human kidney. J. Am. Soc. Nephrol..

[102] Whisenant T.C., Nigam S.K. (2022). Organic Anion Transporters (OAT) and Other SLC22 Transporters in Progression of Renal Cell Carcinoma. Cancers.

[103] Marada V.V., Florl S., Kuhne A., Muller J., Burckhardt G., Hagos Y. (2015). Interaction of human organic anion transporter 2 (OAT2) and sodium taurocholate cotransporting polypeptide (NTCP) with antineoplastic drugs. Pharmacol. Res..

[104] Shen H., Liu T., Morse B.L., Zhao Y., Zhang Y., Qiu X., Chen C., Lewin A.C., Wang X.T., Liu G. (2015). Characterization of Organic Anion Transporter 2 (SLC22A7): A Highly Efficient Transporter for Creatinine and Species-Dependent Renal Tubular Expression. Drug Metab. Dispos..

[105] Cheng Y., Vapurcuyan A., Shahidullah M., Aleksunes L.M., Pelis R.M. (2012). Expression of organic anion transporter 2 in the human kidney and its potential role in the tubular secretion of guanine-containing antiviral drugs. Drug Metab. Dispos..

[106] Breljak D., Ljubojevic M., Hagos Y., Micek V., Balen Eror D., Vrhovac Madunic I., Brzica H., Karaica D., Radovic N., Kraus O. (2016). Distribution of organic anion transporters NaDC3 and OAT1-3 along the human nephron. Am. J. Physiol. Renal. Physiol..

[107] Dahlin A., Geier E., Stocker S.L., Cropp C.D., Grigorenko E., Bloomer M., Siegenthaler J., Xu L., Basile A.S., Tang-Liu D.D. (2013). Gene expression profiling of transporters in the solute carrier and ATP-binding cassette superfamilies in human eye substructures. Mol. Pharm..

[108] Smith K.M., Ng A.M., Yao S.Y., Labedz K.A., Knaus E.E., Wiebe L.I., Cass C.E., Baldwin S.A., Chen X.Z., Karpinski E. (2004). Electrophysiological characterization of a recombinant human Na+-coupled nucleoside transporter (hCNT1) produced in Xenopus oocytes. J. Physiol..

[109] Garcia-Manteiga J., Molina-Arcas M., Casado F.J., Mazo A., Pastor-Anglada M. (2003). Nucleoside transporter profiles in human pancreatic cancer cells: Role of hCNT1 in 2’,2’-difluorodeoxycytidine- induced cytotoxicity. Clin. Cancer Res..

[110] Mini E., Nobili S., Caciagli B., Landini I., Mazzei T. (2006). Cellular pharmacology of gemcitabine. Ann. Oncol..

[111] Huang Q.Q., Harvey C.M., Paterson A.R., Cass C.E., Young J.D. (1993). Functional expression of Na(+)-dependent nucleoside transport systems of rat intestine in isolated oocytes of Xenopus laevis. Demonstration that rat jejunum expresses the purine-selective system N1 (cif) and a second, novel system N3 having broad specificity for purine and pyrimidine nucleosides. J. Biol. Chem..

[112] Anderson C.M., Xiong W., Young J.D., Cass C.E., Parkinson F.E. (1996). Demonstration of the existence of mRNAs encoding N1/cif and N2/cit sodium/nucleoside cotransporters in rat brain. Brain Res. Mol. Brain Res..

[113] Pennycooke M., Chaudary N., Shuralyova I., Zhang Y., Coe I.R. (2001). Differential expression of human nucleoside transporters in normal and tumor tissue. Biochem. Biophys. Res. Commun..

[114] Farre X., Guillen-Gomez E., Sanchez L., Hardisson D., Plaza Y., Lloberas J., Casado F.J., Palacios J., Pastor-Anglada M. (2004). Expression of the nucleoside-derived drug transporters hCNT1, hENT1 and hENT2 in gynecologic tumors. Int. J. Cancer.

[115] Mohelnikova-Duchonova B., Brynychova V., Hlavac V., Kocik M., Oliverius M., Hlavsa J., Honsova E., Mazanec J., Kala Z., Melichar B. (2013). The association between the expression of solute carrier transporters and the prognosis of pancreatic cancer. Cancer Chemother. Pharmacol..

[116] Lang T.T., Selner M., Young J.D., Cass C.E. (2001). Acquisition of human concentrative nucleoside transporter 2 (hcnt2) activity by gene transfer confers sensitivity to fluoropyrimidine nucleosides in drug-resistant leukemia cells. Mol. Pharmacol..

[117] Smith K.M., Slugoski M.D., Cass C.E., Baldwin S.A., Karpinski E., Young J.D. (2007). Cation coupling properties of human concentrative nucleoside transporters hCNT1, hCNT2 and hCNT3. Mol. Membr. Biol..

[118] Che M., Ortiz D.F., Arias I.M. (1995). Primary structure and functional expression of a cDNA encoding the bile canalicular, purine-specific Na(+)-nucleoside cotransporter. J. Biol. Chem..

[119] Guillen-Gomez E., Calbet M., Casado J., de Lecea L., Soriano E., Pastor-Anglada M., Burgaya F. (2004). Distribution of CNT2 and ENT1 transcripts in rat brain: Selective decrease of CNT2 mRNA in the cerebral cortex of sleep-deprived rats. J. Neurochem..

[120] Minuesa G., Purcet S., Erkizia I., Molina-Arcas M., Bofill M., Izquierdo-Useros N., Casado F.J., Clotet B., Pastor-Anglada M., Martinez-Picado J. (2008). Expression and functionality of anti-human immunodeficiency virus and anticancer drug uptake transporters in immune cells. J. Pharmacol. Exp. Ther..

[121] Liu J., Wang D., Zhang C., Zhang Z., Chen X., Lian J., Liu J., Wang G., Yuan W., Sun Z. (2018). Identification of liver metastasis-associated genes in human colon carcinoma by mRNA profiling. Chin. J. Cancer Res..

[122] Sundaram M., Yao S.Y., Ng A.M., Cass C.E., Baldwin S.A., Young J.D. (2001). Equilibrative nucleoside transporters: Mapping regions of interaction for the substrate analogue nitrobenzylthioinosine (NBMPR) using rat chimeric proteins. Biochemistry.

[123] Zhang J., Visser F., King K.M., Baldwin S.A., Young J.D., Cass C.E. (2007). The role of nucleoside transporters in cancer chemotherapy with nucleoside drugs. Cancer Metastasis Rev..

[124] Inoue K. (2017). Molecular Basis of Nucleobase Transport Systems in Mammals. Biol. Pharm. Bull..

[125] Shimada T., Nakanishi T., Tajima H., Yamazaki M., Yokono R., Takabayashi M., Shimada T., Sawamoto K., Miyamoto K., Kitagawa H. (2015). Saturable Hepatic Extraction of Gemcitabine Involves Biphasic Uptake Mediated by Nucleoside Transporters Equilibrative Nucleoside Transporter 1 and 2. J. Pharm. Sci..

[126] Hartmann E., Fernandez V., Moreno V., Valls J., Hernandez L., Bosch F., Abrisqueta P., Klapper W., Dreyling M., Hoster E. (2008). Five-gene model to predict survival in mantle-cell lymphoma using frozen or formalin-fixed, paraffin-embedded tissue. J. Clin. Oncol..

[127] Chen C.F., Hsu E.C., Lin K.T., Tu P.H., Chang H.W., Lin C.H., Chen Y.J., Gu D.L., Lin C.H., Wu J.Y. (2010). Overlapping high-resolution copy number alterations in cancer genomes identified putative cancer genes in hepatocellular carcinoma. Hepatology.

[128] Kim H., Wu X., Lee J. (2013). SLC31 (CTR) family of copper transporters in health and disease. Mol. Aspects Med..

[129] Kilari D., Iczkowski K.A., Pandya C., Robin A.J., Messing E.M., Guancial E., Kim E.S. (2016). Copper Transporter-CTR1 Expression and Pathological Outcomes in Platinum-treated Muscle-invasive Bladder Cancer Patients. Anticancer Res..

[130] Ishida S., Lee J., Thiele D.J., Herskowitz I. (2002). Uptake of the anticancer drug cisplatin mediated by the copper transporter Ctr1 in yeast and mammals. Proc. Natl. Acad. Sci. USA.

[131] Howell S.B., Safaei R., Larson C.A., Sailor M.J. (2010). Copper transporters and the cellular pharmacology of the platinum-containing cancer drugs. Mol. Pharmacol..

[132] Kuo M.T., Chen H.H., Song I.S., Savaraj N., Ishikawa T. (2007). The roles of copper transporters in cisplatin resistance. Cancer Metastasis Rev..

[133] Zhao R., Goldman I.D. (2013). Folate and thiamine transporters mediated by facilitative carriers (SLC19A1-3 and SLC46A1) and folate receptors. Mol. Aspects Med..

[134] Desmoulin S.K., Hou Z., Gangjee A., Matherly L.H. (2012). The human proton-coupled folate transporter: Biology and therapeutic applications to cancer. Cancer Biol. Ther..

[135] Qiu A., Jansen M., Sakaris A., Min S.H., Chattopadhyay S., Tsai E., Sandoval C., Zhao R., Akabas M.H., Goldman I.D. (2006). Identification of an intestinal folate transporter and the molecular basis for hereditary folate malabsorption. Cell.

[136] Qiu A., Min S.H., Jansen M., Malhotra U., Tsai E., Cabelof D.C., Matherly L.H., Zhao R., Akabas M.H., Goldman I.D. (2007). Rodent intestinal folate transporters (SLC46A1): Secondary structure, functional properties, and response to dietary folate restriction. Am. J. Physiol. Cell Physiol..

[137] Urquhart B.L., Gregor J.C., Chande N., Knauer M.J., Tirona R.G., Kim R.B. (2010). The human proton-coupled folate transporter (hPCFT): Modulation of intestinal expression and function by drugs. Am. J. Physiol. Gastrointest. Liver Physiol..

[138] Shayeghi M., Latunde-Dada G.O., Oakhill J.S., Laftah A.H., Takeuchi K., Halliday N., Khan Y., Warley A., McCann F.E., Hider R.C. (2005). Identification of an intestinal heme transporter. Cell.

[139] Gnana-Prakasam J.P., Reddy S.K., Veeranan-Karmegam R., Smith S.B., Martin P.M., Ganapathy V. (2011). Polarized distribution of heme transporters in retinal pigment epithelium and their regulation in the iron-overload disease hemochromatosis. Invest Ophthalmol. Vis. Sci..

[140] Zhao R., Min S.H., Wang Y., Campanella E., Low P.S., Goldman I.D. (2009). A role for the proton-coupled folate transporter (PCFT-SLC46A1) in folate receptor-mediated endocytosis. J. Biol. Chem..

[141] Hlavac V., Vaclavikova R., Brynychova V., Dvorak P., Elsnerova K., Kozevnikovova R., Raus K., Kopeckova K., Mestakova S., Vrana D. (2021). SLC46A1 Haplotype with Predicted Functional Impact has Prognostic Value in Breast Carcinoma. Mol. Diagn. Ther..

[142] Motohashi H., Inui K. (2013). Organic cation transporter OCTs (SLC22) and MATEs (SLC47) in the human kidney. AAPS J..

[143] Terada T., Inui K. (2008). Physiological and pharmacokinetic roles of H+/organic cation antiporters (MATE/SLC47A). Biochem. Pharmacol..

[144] Otsuka M., Matsumoto T., Morimoto R., Arioka S., Omote H., Moriyama Y. (2005). A human transporter protein that mediates the final excretion step for toxic organic cations. Proc. Natl. Acad. Sci. USA.

[145] Dresser M.J., Leabman M.K., Giacomini K.M. (2001). Transporters involved in the elimination of drugs in the kidney: Organic anion transporters and organic cation transporters. J. Pharm. Sci..

[146] Xie J., Xia L., Xiang W., He W., Yin H., Wang F., Gao T., Qi W., Yang Z., Yang X. (2020). Metformin selectively inhibits metastatic colorectal cancer with the KRAS mutation by intracellular accumulation through silencing MATE1. Proc. Natl. Acad. Sci. USA.

[147] Masuda S., Terada T., Yonezawa A., Tanihara Y., Kishimoto K., Katsura T., Ogawa O., Inui K. (2006). Identification and functional characterization of a new human kidney-specific H+/organic cation antiporter, kidney-specific multidrug and toxin extrusion 2. J. Am. Soc. Nephrol..

[148] Komatsu T., Hiasa M., Miyaji T., Kanamoto T., Matsumoto T., Otsuka M., Moriyama Y., Omote H. (2011). Characterization of the human MATE2 proton-coupled polyspecific organic cation exporter. Int. J. Biochem. Cell Biol..

[149] Pizzagalli M.D., Bensimon A., Superti-Furga G. (2021). A guide to plasma membrane solute carrier proteins. FEBS J..

[150] Chen M., Neul C., Schaeffeler E., Frisch F., Winter S., Schwab M., Koepsell H., Hu S., Laufer S., Baker S.D. (2020). Sorafenib Activity and Disposition in Liver Cancer Does Not Depend on Organic Cation Transporter 1. Clin. Pharmacol. Ther..

[151] Swift B., Nebot N., Lee J.K., Han T., Proctor W.R., Thakker D.R., Lang D., Radtke M., Gnoth M.J., Brouwer K.L. (2013). Sorafenib hepatobiliary disposition: Mechanisms of hepatic uptake and disposition of generated metabolites. Drug Metab. Dispos..

[152] Schiliro C., Firestein B.L. (2021). Mechanisms of Metabolic Reprogramming in Cancer Cells Supporting Enhanced Growth and Proliferation. Cells.

[153] Vaupel P., Multhoff G. (2021). Revisiting the Warburg effect: Historical dogma versus current understanding. J. Physiol..

[154] Gillet J.P., Varma S., Gottesman M.M. (2013). The clinical relevance of cancer cell lines. J. Natl. Cancer Inst..

[155] Lee W., Belkhiri A., Lockhart A.C., Merchant N., Glaeser H., Harris E.I., Washington M.K., Brunt E.M., Zaika A., Kim R.B. (2008). Overexpression of OATP1B3 confers apoptotic resistance in colon cancer. Cancer Res..

[156] Haberkorn B., Oswald S., Kehl N., Gessner A., Taudte R.V., Dobert J.P., Zunke F., Fromm M.F., Konig J. (2022). Cancer-type organic anion transporting polypeptide 1B3 (Ct-OATP1B3) is localized in lysosomes and mediates resistance against kinase inhibitors. Mol. Pharmacol..

[157] Czuba L.C., Hillgren K.M., Swaan P.W. (2018). Post-translational modifications of transporters. Pharmacol. Ther..

[158] Schnedl W.J., Ferber S., Johnson J.H., Newgard C.B. (1994). STZ transport and cytotoxicity. Specific enhancement in GLUT2-expressing cells. Diabetes.

[159] Fukumoto H., Seino S., Imura H., Seino Y., Eddy R.L., Fukushima Y., Byers M.G., Shows T.B., Bell G.I. (1988). Sequence, tissue distribution, and chromosomal localization of mRNA encoding a human glucose transporter-like protein. Proc. Natl. Acad. Sci. USA.

[160] Thorens B., Sarkar H.K., Kaback H.R., Lodish H.F. (1988). Cloning and functional expression in bacteria of a novel glucose transporter present in liver, intestine, kidney, and beta-pancreatic islet cells. Cell.

[161] Thorens B., Wu Y.J., Leahy J.L., Weir G.C. (1992). The loss of GLUT2 expression by glucose-unresponsive beta cells of db/db mice is reversible and is induced by the diabetic environment. J. Clin. Investig..

[162] Mueckler M. (1994). Facilitative glucose transporters. Eur. J. Biochem..

[163] Yan R., Zhao X., Lei J., Zhou Q. (2019). Structure of the human LAT1-4F2hc heteromeric amino acid transporter complex. Nature.

[164] Puris E., Gynther M., Auriola S., Huttunen K.M. (2020). L-Type amino acid transporter 1 as a target for drug delivery. Pharm. Res..

[165] Geier E.G., Schlessinger A., Fan H., Gable J.E., Irwin J.J., Sali A., Giacomini K.M. (2013). Structure-based ligand discovery for the Large-neutral Amino Acid Transporter 1, LAT-1. Proc. Natl. Acad. Sci. USA.

[166] Hagenbuch B., Stieger B. (2013). The SLCO (former SLC21) superfamily of transporters. Mol. Aspects Med..

[167] Badagnani I., Castro R.A., Taylor T.R., Brett C.M., Huang C.C., Stryke D., Kawamoto M., Johns S.J., Ferrin T.E., Carlson E.J. (2006). Interaction of methotrexate with organic-anion transporting polypeptide 1A2 and its genetic variants. J. Pharmacol. Exp. Ther..

[168] Bauer M., Matsuda A., Wulkersdorfer B., Philippe C., Traxl A., Ozvegy-Laczka C., Stanek J., Nics L., Klebermass E.M., Poschner S. (2018). Influence of OATPs on Hepatic Disposition of Erlotinib Measured With Positron Emission Tomography. Clin. Pharmacol. Ther..

[169] Windt T., Toth S., Patik I., Sessler J., Kucsma N., Szepesi A., Zdrazil B., Ozvegy-Laczka C., Szakacs G. (2019). Identification of anticancer OATP2B1 substrates by an in vitro triple-fluorescence-based cytotoxicity screen. Arch. Toxicol..

[170] Schulte R.R., Ho R.H. (2019). Organic Anion Transporting Polypeptides: Emerging Roles in Cancer Pharmacology. Mol. Pharmacol..

[171] Gao C.M., Pu Z., He C., Liang D., Jia Y., Yuan X., Wang G., Xie H. (2017). Effect of OATP1B1 genetic polymorphism on the uptake of tamoxifen and its metabolite, endoxifen. Oncol. Rep..

[172] Zimmerman E.I., Hu S., Roberts J.L., Gibson A.A., Orwick S.J., Li L., Sparreboom A., Baker S.D. (2013). Contribution of OATP1B1 and OATP1B3 to the disposition of sorafenib and sorafenib-glucuronide. Clin. Cancer Res..

[173] Lancaster C.S., Sprowl J.A., Walker A.L., Hu S., Gibson A.A., Sparreboom A. (2013). Modulation of OATP1B-type transporter function alters cellular uptake and disposition of platinum chemotherapeutics. Mol. Cancer Ther..

[174] Koepsell H. (2011). Substrate recognition and translocation by polyspecific organic cation transporters. Biol. Chem..

[175] Ciarimboli G. (2011). Role of organic cation transporters in drug-induced toxicity. Expert Opin. Drug Metab. Toxicol..

[176] Okabe M., Szakacs G., Reimers M.A., Suzuki T., Hall M.D., Abe T., Weinstein J.N., Gottesman M.M. (2008). Profiling SLCO and SLC22 genes in the NCI-60 cancer cell lines to identify drug uptake transporters. Mol. Cancer Ther..

[177] Yu F., Zhang T., Guo L., Wu B. (2018). Liver Receptor Homolog-1 Regulates Organic Anion Transporter 2 and Docetaxel Pharmacokinetics. Drug Metab. Dispos..

[178] Kobayashi Y., Ohshiro N., Sakai R., Ohbayashi M., Kohyama N., Yamamoto T. (2005). Transport mechanism and substrate specificity of human organic anion transporter 2 (hOat2 [SLC22A7]). J. Pharm. Pharmacol..

[179] Choudhuri S., Cherrington N.J., Li N., Klaassen C.D. (2003). Constitutive expression of various xenobiotic and endobiotic transporter mRNAs in the choroid plexus of rats. Drug Metab. Dispos..

[180] Buist S.C., Cherrington N.J., Choudhuri S., Hartley D.P., Klaassen C.D. (2002). Gender-specific and developmental influences on the expression of rat organic anion transporters. J. Pharmacol. Exp. Ther..

[181] Sweet D.H., Miller D.S., Pritchard J.B., Fujiwara Y., Beier D.R., Nigam S.K. (2002). Impaired organic anion transport in kidney and choroid plexus of organic anion transporter 3 (Oat3 (Slc22a8)) knockout mice. J. Biol. Chem..

[182] Simonson G.D., Vincent A.C., Roberg K.J., Huang Y., Iwanij V. (1994). Molecular cloning and characterization of a novel liver-specific transport protein. J. Cell Sci..

[183] Young J.D., Yao S.Y., Baldwin J.M., Cass C.E., Baldwin S.A. (2013). The human concentrative and equilibrative nucleoside transporter families, SLC28 and SLC29. Mol. Aspects Med..

[184] Gray J.H., Owen R.P., Giacomini K.M. (2004). The concentrative nucleoside transporter family, SLC28. Pflugers. Arch..

[185] Podgorska M., Kocbuch K., Pawelczyk T. (2005). Recent advances in studies on biochemical and structural properties of equilibrative and concentrative nucleoside transporters. Acta Biochim. Pol..

[186] Kang N., Jun A.H., Bhutia Y.D., Kannan N., Unadkat J.D., Govindarajan R. (2010). Human equilibrative nucleoside transporter-3 (hENT3) spectrum disorder mutations impair nucleoside transport, protein localization, and stability. J. Biol. Chem..

[187] Greenhalf W., Ghaneh P., Neoptolemos J.P., Palmer D.H., Cox T.F., Lamb R.F., Garner E., Campbell F., Mackey J.R., Costello E. (2014). Pancreatic cancer hENT1 expression and survival from gemcitabine in patients from the ESPAC-3 trial. J. Natl. Cancer Inst..

[188] Tsujie M., Nakamori S., Nakahira S., Takahashi Y., Hayashi N., Okami J., Nagano H., Dono K., Umeshita K., Sakon M. (2007). Human equilibrative nucleoside transporter 1, as a predictor of 5-fluorouracil resistance in human pancreatic cancer. Anticancer Res..

[189] Hubeek I., Stam R.W., Peters G.J., Broekhuizen R., Meijerink J.P., van Wering E.R., Gibson B.E., Creutzig U., Zwaan C.M., Cloos J. (2005). The human equilibrative nucleoside transporter 1 mediates in vitro cytarabine sensitivity in childhood acute myeloid leukaemia. Br. J. Cancer.

[190] Aller S.G., Eng E.T., De Feo C.J., Unger V.M. (2004). Eukaryotic CTR copper uptake transporters require two faces of the third transmembrane domain for helix packing, oligomerization, and function. J. Biol. Chem..

[191] De Feo C.J., Aller S.G., Siluvai G.S., Blackburn N.J., Unger V.M. (2009). Three-dimensional structure of the human copper transporter hCTR1. Proc. Natl. Acad. Sci. USA.

[192] Lee J., Pena M.M., Nose Y., Thiele D.J. (2002). Biochemical characterization of the human copper transporter Ctr1. J. Biol. Chem..

[193] Sinani D., Adle D.J., Kim H., Lee J. (2007). Distinct mechanisms for Ctr1-mediated copper and cisplatin transport. J. Biol. Chem..

[194] Kilari D., Guancial E., Kim E.S. (2016). Role of copper transporters in platinum resistance. World J. Clin. Oncol..

[195] Zhao R., Qiu A., Tsai E., Jansen M., Akabas M.H., Goldman I.D. (2008). The proton-coupled folate transporter: Impact on pemetrexed transport and on antifolates activities compared with the reduced folate carrier. Mol. Pharmacol..

[196] Omote H., Hiasa M., Matsumoto T., Otsuka M., Moriyama Y. (2006). The MATE proteins as fundamental transporters of metabolic and xenobiotic organic cations. Trends Pharmacol. Sci..

[197] Koepsell H. (2020). Organic Cation Transporters in Health and Disease. Pharmacol. Rev..

[198] Fujita S., Hirota T., Sakiyama R., Baba M., Ieiri I. (2019). Identification of drug transporters contributing to oxaliplatin-induced peripheral neuropathy. J. Neurochem..

[199] Brouwer K.L.R., Evers R., Hayden E., Hu S., Li C.Y., Meyer Zu Schwabedissen H.E., Neuhoff S., Oswald S., Piquette-Miller M., Saran C. (2022). Regulation of Drug Transport Proteins-From Mechanisms to Clinical Impact: A White Paper on Behalf of the International Transporter Consortium. Clin. Pharmacol. Ther..

[200] Zhou S., Shu Y. (2022). Transcriptional Regulation of Solute Carrier (SLC) Drug Transporters. Drug Metab. Dispos..

[201] Evans R.M., Mangelsdorf D.J. (2014). Nuclear Receptors, RXR, and the Big Bang. Cell.

[202] Honkakoski P., Sueyoshi T., Negishi M. (2003). Drug-activated nuclear receptors CAR and PXR. Ann. Med..

[203] Wen J., Zhao M. (2021). OATP1B1 Plays an Important Role in the Transport and Treatment Efficacy of Sorafenib in Hepatocellular Carcinoma. Dis. Markers.

[204] Zhou M., Wang D., Li X., Cao Y., Yi C., Wiredu Ocansey D.K., Zhou Y., Mao F. (2022). Farnesoid-X receptor as a therapeutic target for inflammatory bowel disease and colorectal cancer. Front. Pharmacol..

[205] Deuschle U., Schuler J., Schulz A., Schluter T., Kinzel O., Abel U., Kremoser C. (2012). FXR controls the tumor suppressor NDRG2 and FXR agonists reduce liver tumor growth and metastasis in an orthotopic mouse xenograft model. PLoS ONE.

[206] Huang X., Wang B., Chen R., Zhong S., Gao F., Zhang Y., Niu Y., Li C., Shi G. (2021). The Nuclear Farnesoid X Receptor Reduces p53 Ubiquitination and Inhibits Cervical Cancer Cell Proliferation. Front Cell Dev. Biol..

[207] Girisa S., Henamayee S., Parama D., Rana V., Dutta U., Kunnumakkara A.B. (2021). Targeting Farnesoid X receptor (FXR) for developing novel therapeutics against cancer. Mol. Biomed..

[208] Murray M., Zhou F. (2017). Trafficking and other regulatory mechanisms for organic anion transporting polypeptides and organic anion transporters that modulate cellular drug and xenobiotic influx and that are dysregulated in disease. Br. J. Pharmacol..

[209] Alam K., Crowe A., Wang X., Zhang P., Ding K., Li L., Yue W. (2018). Regulation of Organic Anion Transporting Polypeptides (OATP) 1B1- and OATP1B3-Mediated Transport: An Updated Review in the Context of OATP-Mediated Drug-Drug Interactions. Int. J. Mol. Sci..

[210] Chen S., Li K., Jiang J., Wang X., Chai Y., Zhang C., Deng Q., Shuai L., Feng K., Ma K. (2020). Low expression of organic anion-transporting polypeptide 1B3 predicts a poor prognosis in hepatocellular carcinoma. World J. Surg. Oncol..

[211] Repa J.J., Mangelsdorf D.J. (2000). The role of orphan nuclear receptors in the regulation of cholesterol homeostasis. Annu. Rev. Cell Dev. Biol..

[212] Laurencikiene J., Ryden M. (2012). Liver X receptors and fat cell metabolism. Int. J. Obes..

[213] Meyer Zu Schwabedissen H.E., Bottcher K., Chaudhry A., Kroemer H.K., Schuetz E.G., Kim R.B. (2010). Liver X receptor alpha and farnesoid X receptor are major transcriptional regulators of OATP1B1. Hepatology.

[214] Lin Z., Xia S., Liang Y., Ji L., Pan Y., Jiang S., Wan Z., Tao L., Chen J., Lin C. (2020). LXR activation potentiates sorafenib sensitivity in HCC by activating microRNA-378a transcription. Theranostics.

[215] Lefebvre P., Benomar Y., Staels B. (2010). Retinoid X receptors: Common heterodimerization partners with distinct functions. Trends Endocrinol. Metab..

[216] Austin G., Holcroft A., Rinne N., Wang L., Clark R.E. (2015). Evidence that the pregnane X and retinoid receptors PXR, RAR and RXR may regulate transcription of the transporter hOCT1 in chronic myeloid leukaemia cells. Eur. J. Haematol..

[217] Gou Q., Gong X., Jin J., Shi J., Hou Y. (2017). Peroxisome proliferator-activated receptors (PPARs) are potential drug targets for cancer therapy. Oncotarget.

[218] Wang L., Giannoudis A., Austin G., Clark R.E. (2012). Peroxisome proliferator-activated receptor activation increases imatinib uptake and killing of chronic myeloid leukemia cells. Exp. Hematol..

[219] Allenby G., Janocha R., Kazmer S., Speck J., Grippo J.F., Levin A.A. (1994). Binding of 9-cis-retinoic acid and all-trans-retinoic acid to retinoic acid receptors alpha, beta, and gamma. Retinoic acid receptor gamma binds all-trans-retinoic acid preferentially over 9-cis-retinoic acid. J. Biol. Chem..

[220] le Maire A., Teyssier C., Balaguer P., Bourguet W., Germain P. (2019). Regulation of RXR-RAR Heterodimers by RXR- and RAR-Specific Ligands and Their Combinations. Cells.

[221] Le Vee M., Jouan E., Stieger B., Fardel O. (2013). Differential regulation of drug transporter expression by all-trans retinoic acid in hepatoma HepaRG cells and human hepatocytes. Eur. J. Pharm. Sci..

[222] Huang C.S., Chen H.W., Lin T.Y., Lin A.H., Lii C.K. (2018). Shikonin upregulates the expression of drug-metabolizing enzymes and drug transporters in primary rat hepatocytes. J. Ethnopharmacol..

[223] Le Vee M., Jouan E., Stieger B., Lecureur V., Fardel O. (2015). Regulation of human hepatic drug transporter activity and expression by diesel exhaust particle extract. PLoS ONE.

[224] Safe S., Cheng Y., Jin U.H. (2017). The Aryl Hydrocarbon Receptor (AhR) as a Drug Target for Cancer Chemotherapy. Curr. Opin. Toxicol..

[225] Safe S., Zhang L. (2022). The Role of the Aryl Hydrocarbon Receptor (AhR) and Its Ligands in Breast Cancer. Cancers.

[226] Zhu P., Zhou K., Lu S., Bai Y., Qi R., Zhang S. (2020). Modulation of aryl hydrocarbon receptor inhibits esophageal squamous cell carcinoma progression by repressing COX2/PGE2/STAT3 axis. J. Cell Commun. Signal..

[227] Wu X.G., Peng S.B., Huang Q. (2012). Transcriptional regulation of breast cancer resistance protein. Yi Chuan.

[228] Mahringer A., Bernd A., Miller D.S., Fricker G. (2019). Aryl hydrocarbon receptor ligands increase ABC transporter activity and protein expression in killifish (Fundulus heteroclitus) renal proximal tubules. Biol. Chem..

[229] Honkakoski P. (2022). Searching for Constitutive Androstane Receptor Modulators. Drug Metab. Dispos..

[230] Bae S.D.W., Nguyen R., Qiao L., George J. (2021). Role of the constitutive androstane receptor (CAR) in human liver cancer. Biochim. Biophys. Acta Rev. Cancer.

[231] Jigorel E., Le Vee M., Boursier-Neyret C., Parmentier Y., Fardel O. (2006). Differential regulation of sinusoidal and canalicular hepatic drug transporter expression by xenobiotics activating drug-sensing receptors in primary human hepatocytes. Drug Metab. Dispos..

[232] Ichihara S., Kikuchi R., Kusuhara H., Imai S., Maeda K., Sugiyama Y. (2010). DNA methylation profiles of organic anion transporting polypeptide 1B3 in cancer cell lines. Pharm. Res..

[233] Imai S., Kikuchi R., Tsuruya Y., Naoi S., Nishida S., Kusuhara H., Sugiyama Y. (2013). Epigenetic regulation of organic anion transporting polypeptide 1B3 in cancer cell lines. Pharm. Res..

[234] Bannister A.J., Kouzarides T. (2011). Regulation of chromatin by histone modifications. Cell Res..

[235] Marmorstein R., Trievel R.C. (2009). Histone modifying enzymes: Structures, mechanisms, and specificities. Biochim. Biophys. Acta.

[236] Miller J.L., Grant P.A. (2013). The role of DNA methylation and histone modifications in transcriptional regulation in humans. Subcell Biochem..

[237] Wang Y., Zhu Q., Hu H., Zhu H., Yang B., He Q., Yu L., Zeng S. (2021). Upregulation of histone acetylation reverses organic anion transporter 2 repression and enhances 5-fluorouracil sensitivity in hepatocellular carcinoma. Biochem. Pharmacol..

[238] Zhu Q., Yu L., Qin Z., Chen L., Hu H., Zheng X., Zeng S. (2019). Regulation of OCT2 transcriptional repression by histone acetylation in renal cell carcinoma. Epigenetics.

[239] Pelis R.M., Suhre W.M., Wright S.H. (2006). Functional influence of N-glycosylation in OCT2-mediated tetraethylammonium transport. Am. J. Physiol. Renal Physiol..

[240] Ciarimboli G., Struwe K., Arndt P., Gorboulev V., Koepsell H., Schlatter E., Hirsch J.R. (2004). Regulation of the human organic cation transporter hOCT1. J. Cell Physiol..

[241] Cetinkaya I., Ciarimboli G., Yalcinkaya G., Mehrens T., Velic A., Hirsch J.R., Gorboulev V., Koepsell H., Schlatter E. (2003). Regulation of human organic cation transporter hOCT2 by PKA, PI3K, and calmodulin-dependent kinases. Am. J. Physiol. Renal Physiol..

[242] Lee W., Ha J.M., Sugiyama Y. (2020). Post-translational regulation of the major drug transporters in the families of organic anion transporters and organic anion-transporting polypeptides. J. Biol. Chem..

[243] Duan G., Walther D. (2015). The roles of post-translational modifications in the context of protein interaction networks. PLoS Comput. Biol..

[244] Korkuc P., Walther D. (2017). Towards understanding the crosstalk between protein post-translational modifications: Homo- and heterotypic PTM pair distances on protein surfaces are not random. Proteins.

[245] Alam K., Farasyn T., Crowe A., Ding K., Yue W. (2017). Treatment with proteasome inhibitor bortezomib decreases organic anion transporting polypeptide (OATP) 1B3-mediated transport in a substrate-dependent manner. PLoS ONE.

[246] Fan Y., You G. (2020). Proteasome Inhibitors Bortezomib and Carfilzomib Stimulate the Transport Activity of Human Organic Anion Transporter 1. Mol. Pharmacol..

[247] Mayati A., Moreau A., Le Vee M., Stieger B., Denizot C., Parmentier Y., Fardel O. (2017). Protein Kinases C-Mediated Regulations of Drug Transporter Activity, Localization and Expression. Int. J. Mol. Sci..

[248] Hayden E.R., Chen M., Pasquariello K.Z., Gibson A.A., Petti J.J., Shen S., Qu J., Ong S.S., Chen T., Jin Y. (2021). Regulation of OATP1B1 Function by Tyrosine Kinase-mediated Phosphorylation. Clin. Cancer Res..

[249] Sun J., Damaraju V.L., Cass C.E., Sawyer M. (2014). Inhibition of nucleoside transporters by tyrosine kinase inhibitors and its effects on chemotherapy efficacy. Cancer Cell Microenviron..

[250] Anderson J.T. (2019). Role of OCTN1 (SLC22A4) in the Disposition of Nucleoside Analogs in AML.

[251] Vaupel P., Kallinowski F., Okunieff P. (1989). Blood flow, oxygen and nutrient supply, and metabolic microenvironment of human tumors: A review. Cancer Res..

[252] Harris A.L. (2002). Hypoxia--a key regulatory factor in tumour growth. Nat. Rev. Cancer.

[253] Semenza G.L. (2014). Oxygen sensing, hypoxia-inducible factors, and disease pathophysiology. Annu. Rev. Pathol..

[254] Wenger R.H., Stiehl D.P., Camenisch G. (2005). Integration of oxygen signaling at the consensus HRE. Sci. STKE.

[255] Quintero M., Mackenzie N., Brennan P.A. (2004). Hypoxia-inducible factor 1 (HIF-1) in cancer. Eur. J. Surg Oncol..

[256] Semenza G.L. (2010). Defining the role of hypoxia-inducible factor 1 in cancer biology and therapeutics. Oncogene.

[257] Soni S., Padwad Y.S. (2017). HIF-1 in cancer therapy: Two decade long story of a transcription factor. Acta Oncol..

[258] Chen C., Pore N., Behrooz A., Ismail-Beigi F., Maity A. (2001). Regulation of glut1 mRNA by hypoxia-inducible factor-1. Interaction between H-ras and hypoxia. J. Biol. Chem..

[259] Macheda M.L., Rogers S., Best J.D. (2005). Molecular and cellular regulation of glucose transporter (GLUT) proteins in cancer. J. Cell Physiol..

[260] Ullah M.S., Davies A.J., Halestrap A.P. (2006). The plasma membrane lactate transporter MCT4, but not MCT1, is up-regulated by hypoxia through a HIF-1alpha-dependent mechanism. J. Biol. Chem..

[261] Casanello P., Torres A., Sanhueza F., Gonzalez M., Farias M., Gallardo V., Pastor-Anglada M., San Martin R., Sobrevia L. (2005). Equilibrative nucleoside transporter 1 expression is downregulated by hypoxia in human umbilical vein endothelium. Circ. Res..

[262] Eltzschig H.K., Abdulla P., Hoffman E., Hamilton K.E., Daniels D., Schonfeld C., Loffler M., Reyes G., Duszenko M., Karhausen J. (2005). HIF-1-dependent repression of equilibrative nucleoside transporter (ENT) in hypoxia. J. Exp. Med..

[263] Sweet R., Paul A., Zastre J. (2010). Hypoxia induced upregulation and function of the thiamine transporter, SLC19A3 in a breast cancer cell line. Cancer Biol. Ther..

[264] Shi C., Wu J.B., Chu G.C., Li Q., Wang R., Zhang C., Zhang Y., Kim H.L., Wang J., Zhau H.E. (2014). Heptamethine carbocyanine dye-mediated near-infrared imaging of canine and human cancers through the HIF-1alpha/OATPs signaling axis. Oncotarget.

[265] Jing X., Yang F., Shao C., Wei K., Xie M., Shen H., Shu Y. (2019). Role of hypoxia in cancer therapy by regulating the tumor microenvironment. Mol. Cancer.

[266] Tiwari A., Tashiro K., Dixit A., Soni A., Vogel K., Hall B., Shafqat I., Slaughter J., Param N., Le A. (2020). Loss of HIF1A From Pancreatic Cancer Cells Increases Expression of PPP1R1B and Degradation of p53 to Promote Invasion and Metastasis. Gastroenterology.

[267] Hays A., Apte U., Hagenbuch B. (2013). Organic anion transporting polypeptides expressed in pancreatic cancer may serve as potential diagnostic markers and therapeutic targets for early stage adenocarcinomas. Pharm. Res..

[268] Zhao H., Wu L., Yan G., Chen Y., Zhou M., Wu Y., Li Y. (2021). Inflammation and tumor progression: Signaling pathways and targeted intervention. Signal. Transduct. Target Ther..

[269] Crusz S.M., Balkwill F.R. (2015). Inflammation and cancer: Advances and new agents. Nat. Rev. Clin. Oncol..

[270] Coussens L.M., Werb Z. (2002). Inflammation and cancer. Nature.

[271] Ma Y., Adjemian S., Mattarollo S.R., Yamazaki T., Aymeric L., Yang H., Portela Catani J.P., Hannani D., Duret H., Steegh K. (2013). Anticancer chemotherapy-induced intratumoral recruitment and differentiation of antigen-presenting cells. Immunity.

[272] Shacter E., Weitzman S.A. (2002). Chronic inflammation and cancer. Oncology.

[273] Le Vee M., Lecureur V., Stieger B., Fardel O. (2009). Regulation of drug transporter expression in human hepatocytes exposed to the proinflammatory cytokines tumor necrosis factor-alpha or interleukin-6. Drug Metab. Dispos..

[274] Le Vee M., Gripon P., Stieger B., Fardel O. (2008). Down-regulation of organic anion transporter expression in human hepatocytes exposed to the proinflammatory cytokine interleukin 1beta. Drug Metab. Dispos..

[275] Swietach P. (2019). What is pH regulation, and why do cancer cells need it?. Cancer Metastasis Rev..

[276] Nozawa T., Imai K., Nezu J., Tsuji A., Tamai I. (2004). Functional characterization of pH-sensitive organic anion transporting polypeptide OATP-B in human. J. Pharmacol. Exp. Ther..

[277] Xing F., Hu Q., Qin Y., Xu J., Zhang B., Yu X., Wang W. (2022). The Relationship of Redox With Hallmarks of Cancer: The Importance of Homeostasis and Context. Front. Oncol..

[278] Ganapathy V., Thangaraju M., Prasad P.D. (2009). Nutrient transporters in cancer: Relevance to Warburg hypothesis and beyond. Pharmacol. Ther..

[279] Medina R.A., Owen G.I. (2002). Glucose transporters: Expression, regulation and cancer. Biol. Res..

[280] Jiang X., Xin H., Ren Q., Gu J., Zhu L., Du F., Feng C., Xie Y., Sha X., Fang X. (2014). Nanoparticles of 2-deoxy-D-glucose functionalized poly(ethylene glycol)-co-poly(trimethylene carbonate) for dual-targeted drug delivery in glioma treatment. Biomaterials.

[281] Jiang X., Xin H., Gu J., Du F., Feng C., Xie Y., Fang X. (2014). Enhanced antitumor efficacy by d-glucosamine-functionalized and paclitaxel-loaded poly(ethylene glycol)-co-poly(trimethylene carbonate) polymer nanoparticles. J. Pharm. Sci..

[282] Shao K., Ding N., Huang S., Ren S., Zhang Y., Kuang Y., Guo Y., Ma H., An S., Li Y. (2014). Smart nanodevice combined tumor-specific vector with cellular microenvironment-triggered property for highly effective antiglioma therapy. ACS Nano.

[283] Patra M., Awuah S.G., Lippard S.J. (2016). Chemical Approach to Positional Isomers of Glucose-Platinum Conjugates Reveals Specific Cancer Targeting through Glucose-Transporter-Mediated Uptake in Vitro and in Vivo. J. Am. Chem. Soc..

[284] Halestrap A.P. (2013). The SLC16 gene family—Structure, role and regulation in health and disease. Mol. Aspects Med..

[285] Pertega-Gomes N., Vizcaino J.R., Miranda-Goncalves V., Pinheiro C., Silva J., Pereira H., Monteiro P., Henrique R.M., Reis R.M., Lopes C. (2011). Monocarboxylate transporter 4 (MCT4) and CD147 overexpression is associated with poor prognosis in prostate cancer. BMC Cancer.

[286] Simoes-Sousa S., Granja S., Pinheiro C., Fernandes D., Longatto-Filho A., Laus A.C., Alves C.D., Suarez-Penaranda J.M., Perez-Sayans M., Lopes Carvalho A. (2016). Prognostic significance of monocarboxylate transporter expression in oral cavity tumors. Cell Cycle.

[287] Afonso J., Pinto T., Simoes-Sousa S., Schmitt F., Longatto-Filho A., Pinheiro C., Marques H., Baltazar F. (2019). Clinical significance of metabolism-related biomarkers in non-Hodgkin lymphoma—MCT1 as potential target in diffuse large B cell lymphoma. Cell Oncol..

[288] Venishetty V.K., Samala R., Komuravelli R., Kuncha M., Sistla R., Diwan P.V. (2013). beta-Hydroxybutyric acid grafted solid lipid nanoparticles: A novel strategy to improve drug delivery to brain. Nanomedicine.

[289] Birsoy K., Wang T., Possemato R., Yilmaz O.H., Koch C.E., Chen W.W., Hutchins A.W., Gultekin Y., Peterson T.R., Carette J.E. (2013). MCT1-mediated transport of a toxic molecule is an effective strategy for targeting glycolytic tumors. Nat. Genet..

[290] Shennan D.B., Thomson J., Gow I.F., Travers M.T., Barber M.C. (2004). L-leucine transport in human breast cancer cells (MCF-7 and MDA-MB-231): Kinetics, regulation by estrogen and molecular identity of the transporter. Biochim. Biophys. Acta.

[291] Puris E., Fricker G., Gynther M. (2022). Targeting Transporters for Drug Delivery to the Brain: Can We Do Better?. Pharm. Res..

[292] Ohshima Y., Suzuki H., Hanaoka H., Sasaki I., Watanabe S., Haba H., Arano Y., Tsushima Y., Ishioka N.S. (2020). Preclinical evaluation of new alpha-radionuclide therapy targeting LAT1: 2-[(211)At]astato-alpha-methyl-L-phenylalanine in tumor-bearing model. Nucl. Med. Biol..

[293] Wang Z., Lin X., Chi D., Xu Z., Lin G., Liu H., Sun J., He Z., Wang Y. (2020). Single-ligand dual-targeting irinotecan liposomes: Control of targeting ligand display by pH-responsive PEG-shedding strategy to enhance tumor-specific therapy and attenuate toxicity. Int. J. Pharm..

[294] Pocasap P., Weerapreeyakul N., Timonen J., Jarvinen J., Leppanen J., Karkkainen J., Rautio J. (2020). Tyrosine-Chlorambucil Conjugates Facilitate Cellular Uptake through L-Type Amino Acid Transporter 1 (LAT1) in Human Breast Cancer Cell Line MCF-7. Int. J. Mol. Sci..

[295] Hosoya K., Kyoko H., Toyooka N., Kato A., Orihashi M., Tomi M., Tachikawa M. (2008). Evaluation of amino acid-mustard transport as L-type amino acid transporter 1 (LAT1)-mediated alkylating agents. Biol. Pharm. Bull..

[296] Hong S., Fang Z., Jung H.Y., Yoon J.H., Hong S.S., Maeng H.J. (2018). Synthesis of Gemcitabine-Threonine Amide Prodrug Effective on Pancreatic Cancer Cells with Improved Pharmacokinetic Properties. Molecules.

[297] Singh V.K., Subudhi B.B. (2016). Development and characterization of lysine-methotrexate conjugate for enhanced brain delivery. Drug Deliv..

[298] Bhunia S., Vangala V., Bhattacharya D., Ravuri H.G., Kuncha M., Chakravarty S., Sistla R., Chaudhuri A. (2017). Large Amino Acid Transporter 1 Selective Liposomes of l-DOPA Functionalized Amphiphile for Combating Glioblastoma. Mol. Pharm..

[299] Ong Z.Y., Chen S., Nabavi E., Regoutz A., Payne D.J., Elson D.S., Dexter D.T., Dunlop I.E., Porter A.E. (2017). Multibranched Gold Nanoparticles with Intrinsic LAT-1 Targeting Capabilities for Selective Photothermal Therapy of Breast Cancer. ACS Appl. Mater. Interfaces.

[300] Fuchs B.C., Bode B.P. (2005). Amino acid transporters ASCT2 and LAT1 in cancer: Partners in crime?. Semin. Cancer Biol..

[301] Zhou P., Liang X., Zhou C., Qin J., Hou C., Zhu Z., Zhang W., Wang S., Zhong D. (2019). Glutamine-beta-cyclodextrin for targeted doxorubicin delivery to triple-negative breast cancer tumors via the transporter ASCT2. J. Mater. Chem. B.

[302] Wang C., Wu J., Wang Z., Yang Z., Li Z., Deng H., Li L., Peng X., Feng M. (2018). Glutamine addiction activates polyglutamine-based nanocarriers delivering therapeutic siRNAs to orthotopic lung tumor mediated by glutamine transporter SLC1A5. Biomaterials.

[303] Sloan J.L., Mager S. (1999). Cloning and functional expression of a human Na(+) and Cl(-)-dependent neutral and cationic amino acid transporter B(0+). J. Biol. Chem..

[304] Gupta N., Miyauchi S., Martindale R.G., Herdman A.V., Podolsky R., Miyake K., Mager S., Prasad P.D., Ganapathy M.E., Ganapathy V. (2005). Upregulation of the amino acid transporter ATB0,+ (SLC6A14) in colorectal cancer and metastasis in humans. Biochim. Biophys. Acta.

[305] Gupta N., Prasad P.D., Ghamande S., Moore-Martin P., Herdman A.V., Martindale R.G., Podolsky R., Mager S., Ganapathy M.E., Ganapathy V. (2006). Up-regulation of the amino acid transporter ATB(0,+) (SLC6A14) in carcinoma of the cervix. Gynecol. Oncol..

[306] Luo Q., Gong P., Sun M., Kou L., Ganapathy V., Jing Y., He Z., Sun J. (2016). Transporter occluded-state conformation-induced endocytosis: Amino acid transporter ATB(0,+)-mediated tumor targeting of liposomes for docetaxel delivery for hepatocarcinoma therapy. J. Control Release.

[307] Luo Q., Yang B., Tao W., Li J., Kou L., Lian H., Che X., He Z., Sun J. (2017). ATB(0,+) transporter-mediated targeting delivery to human lung cancer cells via aspartate-modified docetaxel-loading stealth liposomes. Biomater. Sci..

[308] Kou L., Huang H., Lin X., Jiang X., Wang Y., Luo Q., Sun J., Yao Q., Ganapathy V., Chen R. (2020). Endocytosis of ATB(0,+)(SLC6A14)-targeted liposomes for drug delivery and its therapeutic application for pancreatic cancer. Expert Opin. Drug Deliv..

[309] Spanier B., Rohm F. (2018). Proton Coupled Oligopeptide Transporter 1 (PepT1) Function, Regulation, and Influence on the Intestinal Homeostasis. Compr. Physiol..

[310] Tai W., Chen Z., Cheng K. (2013). Expression profile and functional activity of peptide transporters in prostate cancer cells. Mol. Pharm..

[311] Schniers B.K., Rajasekaran D., Korac K., Sniegowski T., Ganapathy V., Bhutia Y.D. (2021). PEPT1 is essential for the growth of pancreatic cancer cells: A viable drug target. Biochem. J..

[312] Tao W., Zhao D., Sun M., Wang Z., Lin B., Bao Y., Li Y., He Z., Sun Y., Sun J. (2018). Intestinal absorption and activation of decitabine amino acid ester prodrugs mediated by peptide transporter PEPT1 and enterocyte enzymes. Int. J. Pharm..

[313] Thompson B.R., Shi J., Zhu H.J., Smith D.E. (2020). Pharmacokinetics of gemcitabine and its amino acid ester prodrug following intravenous and oral administrations in mice. Biochem. Pharmacol..

[314] Du Y., Tian C., Wang M., Huang D., Wei W., Liu Y., Li L., Sun B., Kou L., Kan Q. (2018). Dipeptide-modified nanoparticles to facilitate oral docetaxel delivery: New insights into PepT1-mediated targeting strategy. Drug Deliv..

[315] Landowski C.P., Vig B.S., Song X., Amidon G.L. (2005). Targeted delivery to PEPT1-overexpressing cells: Acidic, basic, and secondary floxuridine amino acid ester prodrugs. Mol. Cancer Ther..

[316] Gong Y., Wu X., Wang T., Zhao J., Liu X., Yao Z., Zhang Q., Jian X. (2017). Targeting PEPT1: A novel strategy to improve the antitumor efficacy of doxorubicin in human hepatocellular carcinoma therapy. Oncotarget.

[317] Ancey P.B., Contat C., Meylan E. (2018). Glucose transporters in cancer—From tumor cells to the tumor microenvironment. FEBS J..

[318] Chai Y.J., Yi J.W., Oh S.W., Kim Y.A., Yi K.H., Kim J.H., Lee K.E. (2017). Upregulation of SLC2 (GLUT) family genes is related to poor survival outcomes in papillary thyroid carcinoma: Analysis of data from The Cancer Genome Atlas. Surgery.

[319] Younes M., Lechago L.V., Lechago J. (1996). Overexpression of the human erythrocyte glucose transporter occurs as a late event in human colorectal carcinogenesis and is associated with an increased incidence of lymph node metastases. Clin. Cancer Res..

[320] Airley R., Loncaster J., Davidson S., Bromley M., Roberts S., Patterson A., Hunter R., Stratford I., West C. (2001). Glucose transporter glut-1 expression correlates with tumor hypoxia and predicts metastasis-free survival in advanced carcinoma of the cervix. Clin. Cancer Res..

[321] Shan X.H., Hu H., Xiong F., Gu N., Geng X.D., Zhu W., Lin J., Wang Y.F. (2012). Targeting Glut1-overexpressing MDA-MB-231 cells with 2-deoxy-D-g1ucose modified SPIOs. Eur. J. Radiol..

[322] Xiong F., Zhu Z.Y., Xiong C., Hua X.Q., Shan X.H., Zhang Y., Gu N. (2012). Preparation, characterization of 2-deoxy-D-glucose functionalized dimercaptosuccinic acid-coated maghemite nanoparticles for targeting tumor cells. Pharm. Res..

[323] Draoui N., Feron O. (2011). Lactate shuttles at a glance: From physiological paradigms to anti-cancer treatments. Dis. Model Mech..

[324] Dimmer K.S., Friedrich B., Lang F., Deitmer J.W., Broer S. (2000). The low-affinity monocarboxylate transporter MCT4 is adapted to the export of lactate in highly glycolytic cells. Biochem. J..

[325] Halestrap A.P., Meredith D. (2004). The SLC16 gene family-from monocarboxylate transporters (MCTs) to aromatic amino acid transporters and beyond. Pflugers. Arch..

[326] Kim H.K., Lee I., Bang H., Kim H.C., Lee W.Y., Yun S.H., Lee J., Lee S.J., Park Y.S., Kim K.M. (2018). MCT4 Expression Is a Potential Therapeutic Target in Colorectal Cancer with Peritoneal Carcinomatosis. Mol. Cancer Ther..

[327] Lopes-Coelho F., Nunes C., Gouveia-Fernandes S., Rosas R., Silva F., Gameiro P., Carvalho T., Gomes da Silva M., Cabecadas J., Dias S. (2017). Monocarboxylate transporter 1 (MCT1), a tool to stratify acute myeloid leukemia (AML) patients and a vehicle to kill cancer cells. Oncotarget.

[328] Lopes C., Pereira C., Medeiros R. (2021). ASCT2 and LAT1 Contribution to the Hallmarks of Cancer: From a Molecular Perspective to Clinical Translation. Cancers.

[329] Rodriguez C.F., Escudero-Bravo P., Diaz L., Bartoccioni P., Garcia-Martin C., Gilabert J.G., Boskovic J., Guallar V., Errasti-Murugarren E., Llorca O. (2021). Structural basis for substrate specificity of heteromeric transporters of neutral amino acids. Proc. Natl. Acad. Sci. USA.

[330] Kaneda-Nakashima K., Zhang Z., Manabe Y., Shimoyama A., Kabayama K., Watabe T., Kanai Y., Ooe K., Toyoshima A., Shirakami Y. (2021). alpha-Emitting cancer therapy using (211) At-AAMT targeting LAT1. Cancer Sci..

[331] Scalise M., Pochini L., Pingitore P., Hedfalk K., Indiveri C. (2015). Cysteine is not a substrate but a specific modulator of human ASCT2 (SLC1A5) transporter. FEBS Lett..

[332] Yoo H.C., Yu Y.C., Sung Y., Han J.M. (2020). Glutamine reliance in cell metabolism. Exp. Mol. Med..

[333] Bhutia Y.D., Ganapathy V. (2016). Glutamine transporters in mammalian cells and their functions in physiology and cancer. Biochim. Biophys. Acta.

[334] Broer S. (2018). Amino Acid Transporters as Disease Modifiers and Drug Targets. SLAS Discov..

[335] Sikder M.O.F., Yang S., Ganapathy V., Bhutia Y.D. (2017). The Na(+)/Cl(-)-Coupled, Broad-Specific, Amino Acid Transporter SLC6A14 (ATB(0,+)): Emerging Roles in Multiple Diseases and Therapeutic Potential for Treatment and Diagnosis. AAPS J..

